# Assessment of Two Restraint Potentials for Coarse-Grained Chemical-Cross-Link-Assisted Modeling of Protein Structures

**DOI:** 10.1021/acs.jcim.3c01890

**Published:** 2024-02-12

**Authors:** Mateusz Leśniewski, Maciej Pyrka, Cezary Czaplewski, Nguyen Truong Co, Yida Jiang, Zhou Gong, Chun Tang, Adam Liwo

**Affiliations:** † Faculty of Chemistry, University of Gdańsk, Fahrenheit Union of Universities, ul. Wita Stwosza 63, 80-308 Gdańsk, Poland; ‡ Department of Physics and Biophysics, University of Warmia and Mazury, ul. Oczapowskiego 4, 10-719 Olsztyn, Poland; ¶ College of Chemistry and Molecular Engineering & Center for Quantitative Biology & PKU-Tsinghua Center for Life Sciences & Beijing National Laboratory for Molecular Sciences, Peking University, Beijing 100871, China; § Innovation Academy of Precision Measurement Science and Technology, Chinese Academy of Sciences, 30 W. Xiao Hong Shan, Wuhan 430071, China

## Abstract

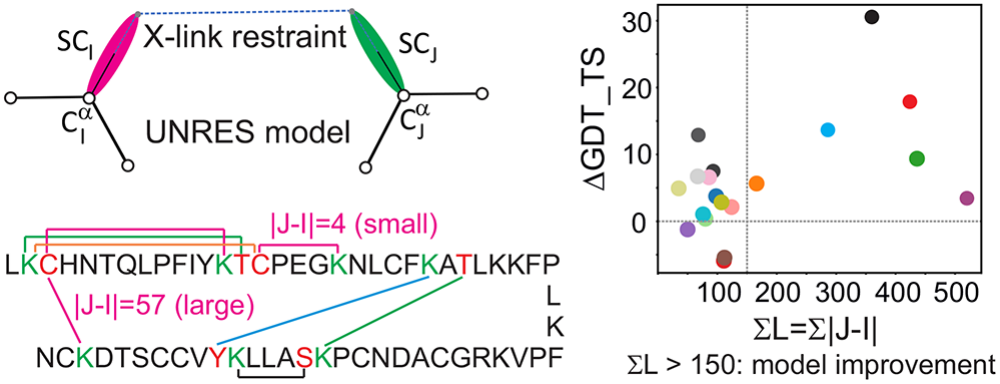

The influence of distance restraints from chemical cross-link mass spectroscopy (XL-MS) on the quality of protein structures modeled with the coarse-grained UNRES force field was assessed by using a protocol based on multiplexed replica exchange molecular dynamics, in which both simulated and experimental cross-link restraints were employed, for 23 small proteins. Six cross-links with upper distance boundaries from 4 Å to 12 Å (azido benzoic acid succinimide (ABAS), triazidotriazine (TATA), succinimidyldiazirine (SDA), disuccinimidyl adipate (DSA), disuccinimidyl glutarate (DSG), and disuccinimidyl suberate (BS^3^)) and two types of restraining potentials ((i) simple flat-bottom Lorentz-like potentials dependent on side chain distance (all cross-links) and (ii) distance- and orientation-dependent potentials determined based on molecular dynamics simulations of model systems (DSA, DSG, BS^3^, and SDA)) were considered. The Lorentz-like potentials with properly set parameters were found to produce a greater number of higher-quality models compared to unrestrained simulations than the MD-based potentials, because the latter can force too long distances between side chains. Therefore, the flat-bottom Lorentz-like potentials are recommended to represent cross-link restraints. It was also found that significant improvement of model quality upon the introduction of cross-link restraints is obtained when the sum of differences of indices of cross-linked residues exceeds 150.

## Introduction

Chemical cross-linking coupled with mass spectrometry (XL-MS) is a relatively inexpensive and fast experimental technique, which furnishes the information on the distances between cross-linkable amino acid residues in proteins that can be used as distance restraints in data-assisted modeling of protein structures.^1−7^ In the XL-MS experiments, a chemical cross-linking reagent, which binds to two groups (usually amino acid side chains) is introduced into the protein solution. When the chemical reaction is complete, the cross-linked protein is digested, this process resulting in cross-linked pairs of oligopeptide fragments excised from the protein. The mixture is analyzed by mass spectrometry to determine which residues have been cross-linked. The information on the distances between these cross-linked residues—in particular, the upper distance boundaries—can be derived from the chemical structure of the cross-linker(s). The cross-linking reagents can be nonspecific^1,2^ or specific with respect to residue type.^3,4,8^

Because the cross-linking experiments are relatively fast and inexpensive, many molecular-modeling software packages use the cross-link information in data-assisted modeling of proteins, protein conformational ensembles,^9^ or protein complexes,^10,11^ or for protein–peptide and protein–protein docking.^8,11−14^ These packages are based on the existing software developed for modeling the structures of proteins or protein complexes such as XPLOR-NIH,^15^ ROSETTA,^16^ MEDUSA,^12^ I-TASSER,^17^ and UNRES,^18,19^ or for protein docking, such as HADDOCK.^20^ Other software for cross-link-assisted protein docking have also been developed.^21^ The methods available for cross-link-assisted modeling are summarized in a number of review articles.^22−24^

The cross-link restraints are imposed on the distances between the α-carbon (C^α^) atoms of the residues involved^3,4,25,26^ or on the distances between side chain ends.^8,9,27^ Restraints from short cross-links imposed on side-chain ends are more precise.^9^ Moreover, the side-chain distances corresponding to short cross-links are well-correlated^28,29^ with the solvent-accessible surface distance (SASD; the shortest path between two amino acid residues without penetrating the solvent-accessible surface of a protein),^28,30^ thus conforming with the condition that only exposed residues can be cross-linked.

Several types of restraining potentials were designed for cross-link-assisted modeling. The most common and simplest to implement are the flat-bottom potentials with upper distance boundary. Restraint potentials of this type were implemented in early applications, in which nonspecific cross-links were used^1,2,26^ and are still used with specific cross-links.^4,8,9,26^ The other ones are statistical pseudopotentials^26^ derived based on cross-link-distance distributions of specific residue pairs obtained from the cross-linking experiments of proteins with known structures.^3^ Recently, we developed pseudopotentials dependent on side-chain–side-chain distance and orientation for cross-link-assisted modeling based on all-atom molecular-dynamics (MD) simulations of the respective cross-link moieties.^27^

Introducing C^α^-distance restraints from loose nonspecific cross-links did not result in significant model improvement, compared to unrestrained simulations.^1,2,26^ Apart from comparatively low confidence of nonspecific cross-links, such cross-links enable us to set only a large distance boundary in restraining potentials (24–30 Å), which could contribute to nonsatisfactory model-quality improvement. The use of specific cross-link information with tighter restraints on the C^α^-distances resulted in remarkable improvement of model quality.^4,26,27^ The quality of structures modeled with the use of cross-link information is expected to increase when short cross-links are used. One kind are those based on bicarboxylic acids with short hydrocarbon chains (e.g., the glutaric or adipic acid) that bridge a lysine side chain or an N-terminal amino group with another one.^9^ Another kind are those based on heterobifunctional cross-linking reagents, which bind to a lysine side chain or an N-terminal amino group with the reactive-ester site and to a side chain of another kind with the photoactive site.^7,31^ With such cross-link restraints and with the use of the ROSETTA^16^ or MEDUSA^12^ force fields and conformational-space search engines, very good results were obtained.^8^

In our recent work,^27^ we introduced the restraining pseudopotentials corresponding to cross-linking lysine side chains with the glutaric (DSG or BS^2^G) or suberic acid (BS^3^), as well as those corresponding to cross-linking glutamic- and aspartic-acid side chains with adipic- (ADH) or pimelic-acid hydrazide (PDH). The potentials were determined by all-atom MD simulations of the respective model systems, and analytical expressions dependent on both distance and orientation of the side-chain ends were fitted to the obtained potentials of mean force. We implemented them in the coarse-grained UNRES model of polypeptide chains developed in our laboratory^18,19,32^ and, later,^14^ in the UNRES web server.^33^ Because of substantial reduction of the number of interaction sites (only two sites per residue), UNRES is able to search the conformational space efficiently, providing an ∼1000-fold extension of the time-scale of simulations, compared to all-atom models.^34^ We tested the longest (BS^3^) cross-link restraints, using both simulated and experimental data, and compared the results with those obtained with the statistical C^α^-distance potentials determined based on the cross-link data of Leitner and colleagues.^3^ We found that the more-sophisticated MD-based restraining potentials performed slightly better than the statistical potentials but, overall, the improvement of model quality was moderate with both types of potentials.

**Figure 1:**
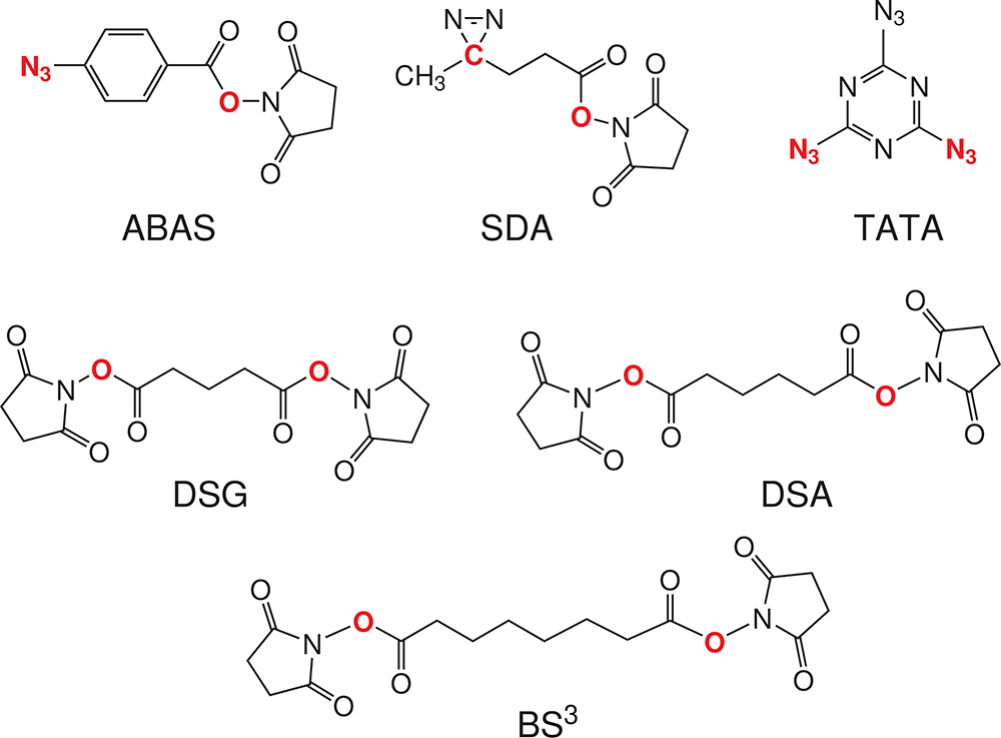
Chemical structures of the cross-linking reagents referenced in this work: azido benzoic acid succinimide (ABAS), succinimidyldiazirine (SDA), triazidotriazine (TATA), disuccinimidyl glutarate (DSG), disuccinimidyl adipate (DSA), and disuccinimidyl suberate (BS^3^). The atoms or groups that are replaced by side-chain/backbone components upon cross-linking are shown in boldface red font. Note that only one of three possible pairs of groups is marked for TATA.

In this work, we extended the cross-link-assisted modeling capacity of UNRES by adding short-distance cross-link restraints. We introduced another lysine-binding homobifunctional cross-linking reagent, disuccinimidyl adipate (DSA), and three heterobifunctional cross-linking reagents—namely, azido benzoic acid succinimide (ABAS), triazidotriazine (TATA), and succinimidyldiazirine (SDA). We also tested the DSG and BS^3^ cross-link potentials determined in our earlier work.^27^ These cross-linking reagents and their use in cross-linking experiments are described in refs (8) and (31). Their chemical structures are shown in Figure 1. The upper distance boundaries range from 4 Å (TATA) to 12 Å (BS^3^). For DSA and SDA, we determined the MD-based potentials and compared their performance, as well as that of DSG, which was determined previously^27^ with the performance of simple flat-bottom Lorentz-like potentials. We found that the latter results in better model quality.

## Methods

### UNRES Model of Polypeptide Chains

**Figure 2:**
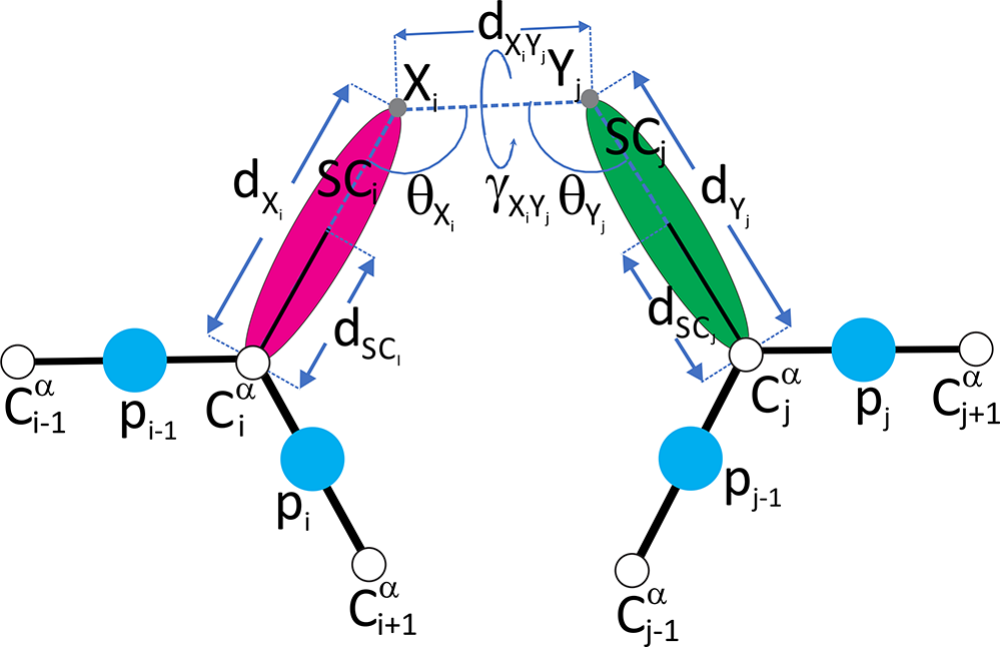
Scheme of the representation of cross-link restraints between residues with indices *i* and *j*, respectively, in the UNRES model. The C^α^ atoms are shown as white spheres, the united side chains (SC) are shown as colored spheroids, and the united peptide groups (p) are shown as blue spheres. The cross-linkable side chains are linked with the appropriate cross-linking reagent. The link is anchored in (approximately) the positions of the side chain atoms that are attached to the cross-link segment. The anchor points (indicated with “X” and “Y”, respectively, and light-gray spheres) are located on the C^α^···SC axes of the UNRES residues. The geometric parameters on which the respective pseudopotentials depend (eqs 3–6) are also shown in the Figure. [Adapted with permission from ref (27). Copyright 2021, John Wiley and Sons.]

UNRES^18,19^ is a heavily coarse-grained model of polypeptide chains, in which the geometry of the polypeptide backbone is defined by the positions of the α-carbon (C^α^) atoms, which are not interaction sites (Figure 2). The interaction sites are united peptide groups, each of which is placed in the middle between the two consecutive C^α^ atoms, and united side chains attached to the respective C^α^ atoms. The coordinates used in the latest implementation of the model^34^ are the Cartesian coordinates of the C^α^ atoms and those of the side chain centers. The energy function is described elsewhere.^18,19^ In this work, we used the NEWCT-9P variant of the UNRES force field calibrated with a set of nine proteins with different structural classes.^32^

The conformational-search engine is molecular dynamics (MD), usually run in the Langevin mode, which has been implemented in UNRES.^35,36^ To make the conformational search more efficient, the multiplexed replica exchange molecular dynamics (MREMD) algorithm^37^ has been implemented.^38^ The MD/MREMD implementation of UNRES has been parallelized^39^ and heavily optimized, including porting to graphical processor units (GPUs).^34,40^

### Cross-Link Restraints with UNRES

Restraints are included in the UNRES energy function in the form of penalty terms. In this study, apart from the cross-link potentials, we imposed the restraints on the C^α^···C^α^···C^α^···C^α^ backbone virtual-bond dihedral angles (γ) in part of the calculations. The extended energy function, including the penalty terms, is given by eq 1.
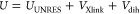
1where *U*_UNRES_ is the UNRES energy function, *V*_Xlink_ the cross-link-penalty term, and *V*_dih_ the dihedral-angle penalty term.

The dihedral-angle restraint potential is defined by eq 2.^41,42^
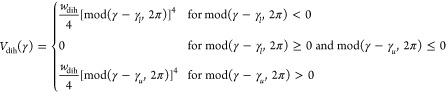
2where γ_*l*_ = 30°, γ_*u*_ = 70° to restrain a virtual-bond dihedral angle to a helical conformation and γ_*l*_ = 120°, γ_*u*_ = 240° to restrain γ to an extended conformation. The weight of the dihedral-angle-restraint term was *w*_dih_ = 50 kcal/(mol rad^4^). These restraints were used in the simulations carried out for the proteins for which experimental cross-link data were used (human serum albumin domains and horse myoglobin).

Because of their coarse-grained nature, the cross-link restraints are straightforward to implement in the UNRES model. In this study, as in our earlier one,^27^ we used fitted potentials of mean force imposed on the distance and orientation of extended united side chains developed based on all-atom molecular dynamics simulations (see the section entitled “Determination of MD-Based Cross-Link Restraining Potentials”), the statistical potentials introduced in refs (26) and (27), which are based on the distributions of the C^α^-distances determined by Leitner and co-workers,^3^ and the flat-bottom Lorentz-like bounded restraining potentials introduced in our earlier work^43^ to handle contact-distance restraints, which we imposed on the distances between the united side chain centers. These variants of the cross-link penalty function will be referred to as the MD-based, statistical, and Lorentz-like potentials and denoted by *V*_Xlink_^MD^, *V*_Xlink_^statis^, and *V*_Xlink_^Lor^, respectively. The respective functional forms are defined and discussed in the remainder of this section.

The MD-based cross-link penalty function is defined by eq 3, with components defined by eqs 4–6.
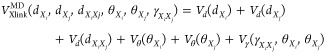
3
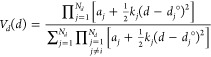
4
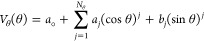
5
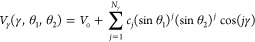
6where *d*_*X*_*i*__ and *d*_*X*_*j*__ are the C^α^···X_*i*_ and C^α^···X_*j*_ virtual-bond lengths, respectively; *d*_*X*_*i*_*X*_*j*__ is the length of the virtual bond linking the terminal cross-link points (which are off the UNRES SC centers but are on the lines pointing from C^α^ to SC); θ_*X*_*i*__ and θ_*X*_*j*__ are the C^α^_*i*_···X_*i*_···X_*j*_ and C^α^_*j*_···X_*j*_···X_*i*_ virtual-bond angles, respectively; γ_*X*_*i*_*X*_*j*__ is the C^α^_*i*_···X_*i*_···X_*j*_···C^α^_*j*_ virtual-bond dihedral angle, while *N*_*d*_, *N*_θ_, and *N*_γ_ are the numbers of terms in the expressions for the virtual-bond-length, virtual-bond-angle, and virtual-bond-dihedral-angle potentials, respectively. The geometric parameters mentioned above are visualized in Figure 2.

The statistical cross-link restraining potentials^3,26,27^ are expressed by eq 7.
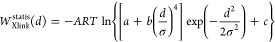
7where *d* is the distance between the C^α^ atoms of the cross-linked residues, *a*, *b*, *c*, and σ are cross-link-specific parameters. *R* is the universal gas constant, and *T* is the absolute temperature; we assumed *T* = 298 K, hence, *RT* = 0.591, and *A* is the weight of the potential, which is assigned the confidence of the cross-link. In this study, we set *A* = 15.

The Lorentz-like cross-link potentials are expressed by eq 8.
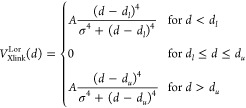
8where *d* is the distance between the side-chain centers from the UNRES structure, *d*_*l*_ and *d*_*u*_ are the lower and upper contact-distance boundaries, respectively, σ is the extent of the restraint-potential slope (wall thickness), and *A* is the restraint-potential well depth. The penalty function has the upper boundary *A*, a feature that results in zero gradient if a restraint is grossly violated. This feature is important if restraints are incorrect in part.

In this work, we set *d*_*l*_ = 2.5 Å, while *d*_*u*_ depended on cross-link type. Four sets of σ and *A* parameters were tried: σ = 5 Å, *A* = 8 kcal/mol; σ = 15 Å, *A* = 8 kcal/mol; σ = 5 Å, *A* = 20 kcal/mol; and σ = 15 Å, *A* = 20 kcal/mol.

### Determination of MD-Based Cross-Link Restraining Potentials

**Figure 3:**
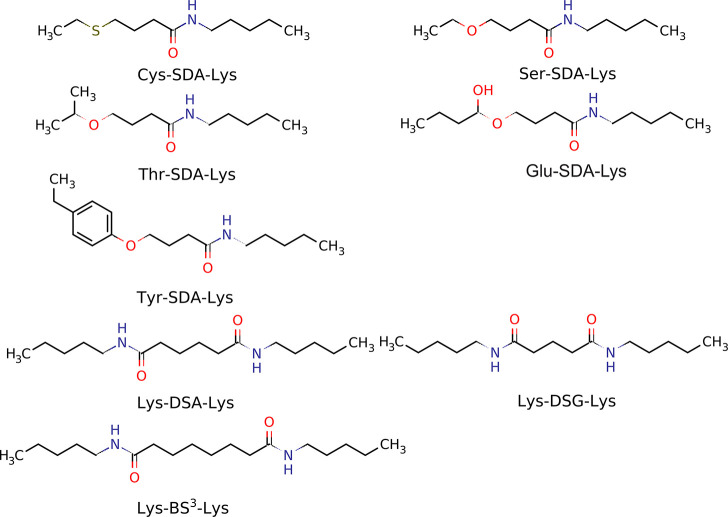
Structures of the compounds modeling the SDA-cross-linked pairs for the derivation of MD-based cross-link potentials introduced in this work and in ref (27). The abbreviations of cross-linking reagents and those of the residues they bridge are shown in each panel.

For all cross-linkers considered in this study (Figure 1), we used the Lorentz-like flat-bottom restraining potential defined by eq 8. The upper flat-bottom boundaries (*d*_*u*_ in eq 8) were equal to 4 Å for TATA, 5 Å for SDA, 6 Å for ABAS and DSG (BS^2^G), 7 Å for DSA, and 12 Å for BS^3^, respectively, according to the maximum dimension of the respective cross-linking-reagent molecule.^8^ The MD-based potentials determined in our previous work^27^ were used for the Lys-DSG-Lys and Lys-BS^3^-Lys cross-links, while those for Lys-DSA-Lys were determined in this work. Of the photoreactive cross-linkers, detailed binding-reaction modes are known only for SDA with serine, cysteine, methionine, threonine, and glutamic acid, respectively; consequently, the MD-based restraining potentials could be determined and used only for those pairs. The respective model compounds are shown in Figure 3.

The MD-based potentials for the DSG and BS^3^ cross-links were determined in our previous work.^27^ Using a similar procedure based on all-atom MD simulations, we determined the parameters for the other cross-link systems shown in Figure 3. The procedure consisted of (i) preparing the respective model systems, including the assignment and determination (if necessary) of force-field parameters, (ii) all-atom MD simulations with explicit water molecules preceded by relaxation and equilibration steps, (iii) calculation of histograms of the respective geometric parameters and, subsequently, of the respective potentials of mean force, and (iv) fitting eqs 4–6 to the determined potentials of mean force.

The MD simulations were carried out by using the AMBER21 package^44^ with the ff19SB force field^45^ and TIP3P water.^46^ The duration of the production phase of the simulations was 2 ns. The structures of the second half of the trajectory (a total of 5000 snapshots) were saved for the calculations of the histograms. Partial atomic charges had to be determined for the compounds modeling SDA-based cross-links, which was performed as follows. First, the structures of model cross-linked systems were constructed (including the C^α^ atoms, which are part of united side chains in UNRES) by using the Gaussview program of the Gaussian16 package.^47^ Subsequently, the structures were energy-minimized by using density functional theory (DFT) with the B3LYP/6-31G* functional, as implemented in the Gaussian-16 program suite. Each optimized structure was subjected to a single-point HF/6-31G* calculation to compute the molecular electrostatic potential around the molecule and the charges were determined by fitting to the electrostatic potential with the RESP procedure^48^ of the ANTECHAMBER module of the AMBER21 package.^44^ The charges are shown in Figure S1 in the Supporting Information.

The histograms in *d*_*X*_*i*_*X*_*j*__, θ_*X*_*i*__, θ_*X*_*j*__, and γ_*X*_*i*_*X*_*j*__ were determined by using the ptraj program of the AMBER21 package and the respective potentials of mean force were calculated, as given by eq 9.
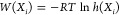
9where *X*_*i*_ is the value of the respective variable at the midpoint of the *i*th bin, *W*(*X*_*i*_) is the potential of mean force corresponding to the *i*th bin, *h*(*X*_*i*_) is the value of the histogram, *R* is the universal gas constant, and *T* is the absolute temperature; we set *T* = 300 K, as in the MD simulations.

The parameters of the analytical formulas (eqs 4–6) were obtained by least-squares fitting of these formulas to the PMFs, by using the Marquardt nonlinear least-squares algorithm.^49^ These parameters are collected in Tables S1–S3 in the Supporting Information and the plots of the fitted restraining potentials superposed on the respective MD-determined PMFs (eq 9) are shown in Figures S1–S7 in the Supporting Information.

### Benchmark Proteins and Simulation Procedure

We used both synthetic and experimental cross-link data to determine the effect of cross-links on the modeled structures. The synthetic data pertained to 12 small single-chain proteins with different structural classes. Their PDB IDs, basic secondary-structure types, chain lengths, as well as the cross-link distances calculated from the experimental structures, are summarized in Table S4 in the Supporting Information. Eight of these proteins (1CLB, 2EM7, 2HNS, 2I09, 1E68, 1KOY, 2FMR, and 1TIG) belong to the set of 69 benchmark proteins that we used to test the current scale-consistent version of UNRES.^32^ UNRES produces reasonably good models of these proteins, except for packing details. The remaining four proteins (1BF0, 1CVO, 1GF4, and 1RXR) were selected based on the presence of a considerable number of cross-linkable residues. The cross-linkable pairs were determined based on the sufficiently small distances between the side chains involved and the location of the potentially cross-linkable side chains on the surface. An additional set of seven benchmark proteins of our previous work,^27^1A6S, 1BG8, 1K40, 1HRE, 1IYU, 1UBQ, and 1VIG, was also used to evaluate the performance of the Lorentz-like cross-link potentials for the longer (BS^3^) cross-links. With this benchmark set, we previously compared the performance of the statistical potentials with that of the MD-based potentials.^27^ The respective cross-links are listed in Table S5 in the Supporting Information. No restraints were imposed on the backbone virtual-bond dihedral angles (γ) in the simulations for the systems mentioned above.

Two proteins, for which the cross-link data pertaining to the ABAS, DSA DSG, SDA, and TATA short cross-linking reagents considered in this work are available—namely, human serum albumin (PDB: 1AO6)^4^ and horse myoglobin (PDB: 2V1H)^8^—were selected. Because of the large size of human serum albumin preventing template-free modeling, we considered repeats 1, 2, 3, and 6 of this protein, for which there were sufficient cross-link restraints, as separate systems. All these four repeats have chain length of ∼100 residues, different cross-link topology and UNRES without cross-link restraints models them with a different quality, thus providing a good basis for the assessment of the impact of cross-link restraints on model quality. Note that 1AO6 also contains disulfide bridges, which were considered as restraints. The Lorentz-like potential (eq 8) was imposed on the distances between the side chains of disulfide-bonded cysteine residues with *d*_*l*_ = 2.5 Å, *d*_*u*_ = 4.5 Å, σ = 5 Å, and *A* = 10 kcal/mol. In summary, five systems with experimental cross-link data were considered. The small size of the systems enabled us to carry out an extensive conformational search, thus reducing the possibility of insufficient sampling.

The experimental cross-link positions are collected in Table S6 in the Supporting Information. It can be seen from the table that, for 2V1H, 9 out of 20 cross-links occur between the residues with C^α^ distances more than 5 Å greater than the maximum length of the respective cross-link; for 3 out of those 9, the distance is more than 10 Å greater. This means that the cross-linking reagents could capture such residue pairs only due to large fluctuations or major distortion of the native conformation. From the point of view of modeling, such restraints are false restraints. To a lesser extent, violations are also present in the first and the second repeat of 1AO6. Because there is no way to tell false cross-link restraints from true cross-link restraints if the structure is unknown, we did not curate these cross-link data to test the robustness of the method. In our earlier work,^43^ we showed that even up to 50% of false distance restraints do not influence the model quality remarkably, provided that the number of restraints is sufficiently large.

Because the proteins mentioned above are of moderate sizes and the objective was mainly to find out how the limited cross-link restraints can help to pack the elements of the structures correctly, we imposed flat-bottom restraints^41,42^ (see eq 2) on the backbone virtual-bond dihedral angles of the helical and extended-strand segments. These segments were assigned according to the HELIX and SHEET records from the respective PDB files.

To model the structures of the benchmark proteins subject to cross-link restraints, we used our four-stage UNRES-based protocol,^41^ which was applied by the UNRES-based prediction groups in the Community Wide Experiments on the Critical Assessment of Techniques for Protein Structure Prediction (CASP).^50^

In stage 1, MREMD simulations were run, using the recently developed optimized version of the UNRES package.^34^ The replicas were run at the following 12 temperatures: 260, 262, 266, 271, 276, 282, 288, 296, 304, 315, 333, and 370 K, respectively, which were selected by using the Hansmann algorithm^51^ to maximize the walks in temperature space. Four replicas were run at a given temperature, giving a total of 48 replicas. Each replica consisted of 20 000 000 time steps, with a step length of 4.89 fs. This value is 0.1 of the “natural MD time unit”, which was introduced in our earlier work^35^ to correspond to expressing energies in kcal/mol and distances in ångströms. The temperatures were exchanged between replicas every 10 000 time steps. The temperature was controlled by the Langevin thermostat, with scaling down the water friction by a factor or 0.01, as in our earlier work.^36^ A modified variable-time-step (VTS)^35^ velocity–Verlet integrator^52^ was used to integrate the equations of motion. The UNRES coordinates were saved every 10 000 time steps, i.e., every replica-exchange time. The last 1000 structures from each trajectory (48 000 structures total) were taken for further analysis.

In stage 2, the structures resulting from MREMD simulations were subjected to post-processing with the UNRES implementation^53^ of the binless weighted histogram analysis method (WHAM)^54^ to enable us to compute the statistical weights of each conformation at any temperature within the replica-temperature range.

In stage 3, the conformational ensembles at *T* = 260, 280, 300, and 330 K (determined by using the information from WHAM to comprise 99% of conformations at a given temperature^53^) were subjected to a cluster analysis with Ward’s minimum variance method.^55^ The number of clusters (and, thereby, the number of models) was set at 5, this number being selected after the rules of CASP,^50^ in which five models per target can be submitted for assessment. The families (and, consequently, the selected structures) were ranked by the cumulative probabilities of all conformations belonging to them, as described in our earlier work.^53^ The structure with the lowest cross-link violation was selected as the representative of a given family.

In stage 4, the coarse-grained models were converted to all-atom models, by using the PULCHRA^56^ and SCWRL^57^ algorithms and refined with AMBER,^58^ as described in our earlier work.^42^

## Results and Discussion

### Synthetic Cross-Link Data

**Figure 4:**
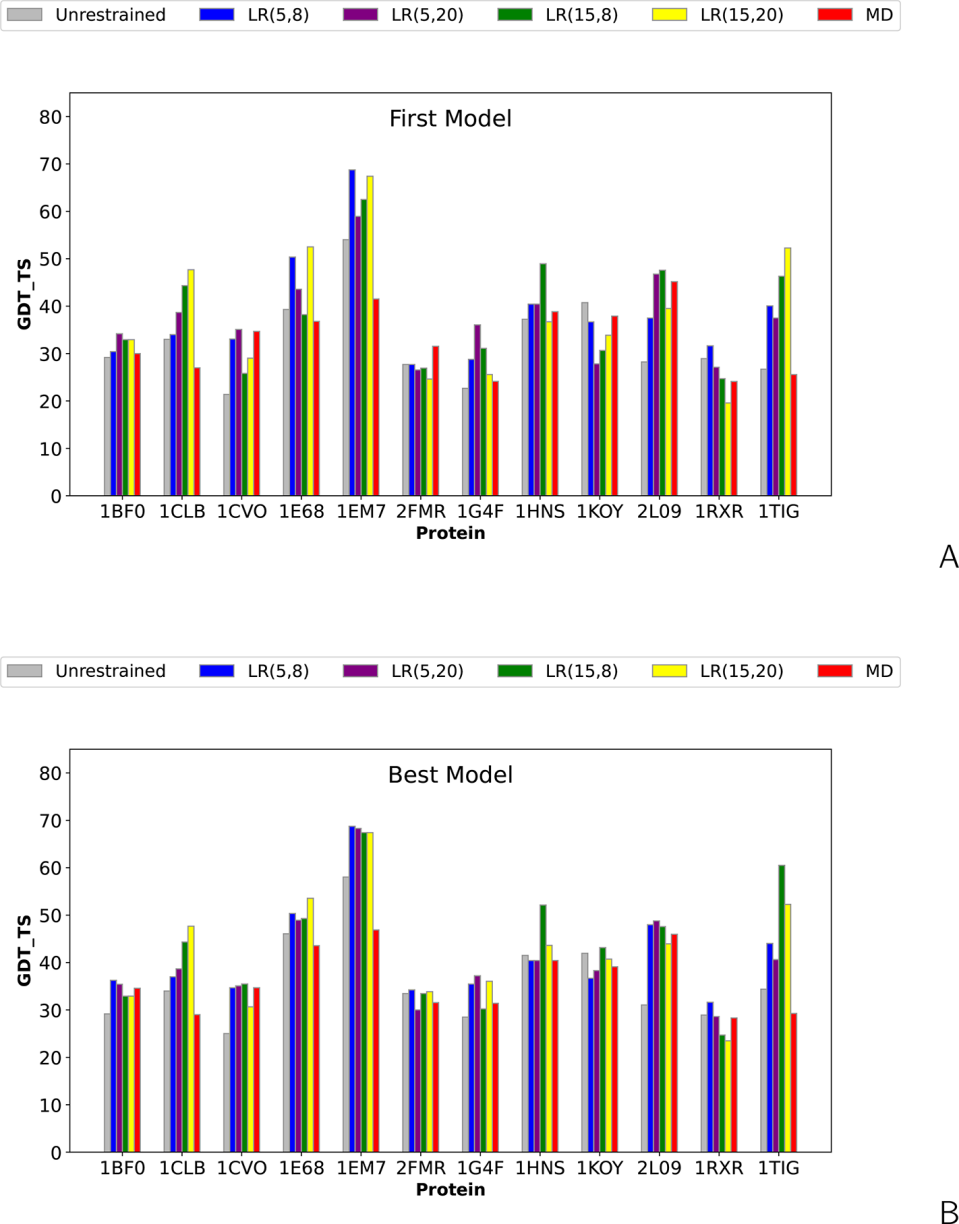
Bar plots of the global distance test total score (GDT_TS) of the (A) first and (B) highest-GDT_TS models of the short-cross-link benchmark proteins obtained in unrestrained UNRES simulations and cross-link-restrained simulations. *LR*(σ,*A*) denotes Lorentz-like potentials, with σ and *A* being the wall thickness and well depth, respectively (eq 8), and MD denotes MD-based potentials (eqs 3–6).

**Figure 5:**
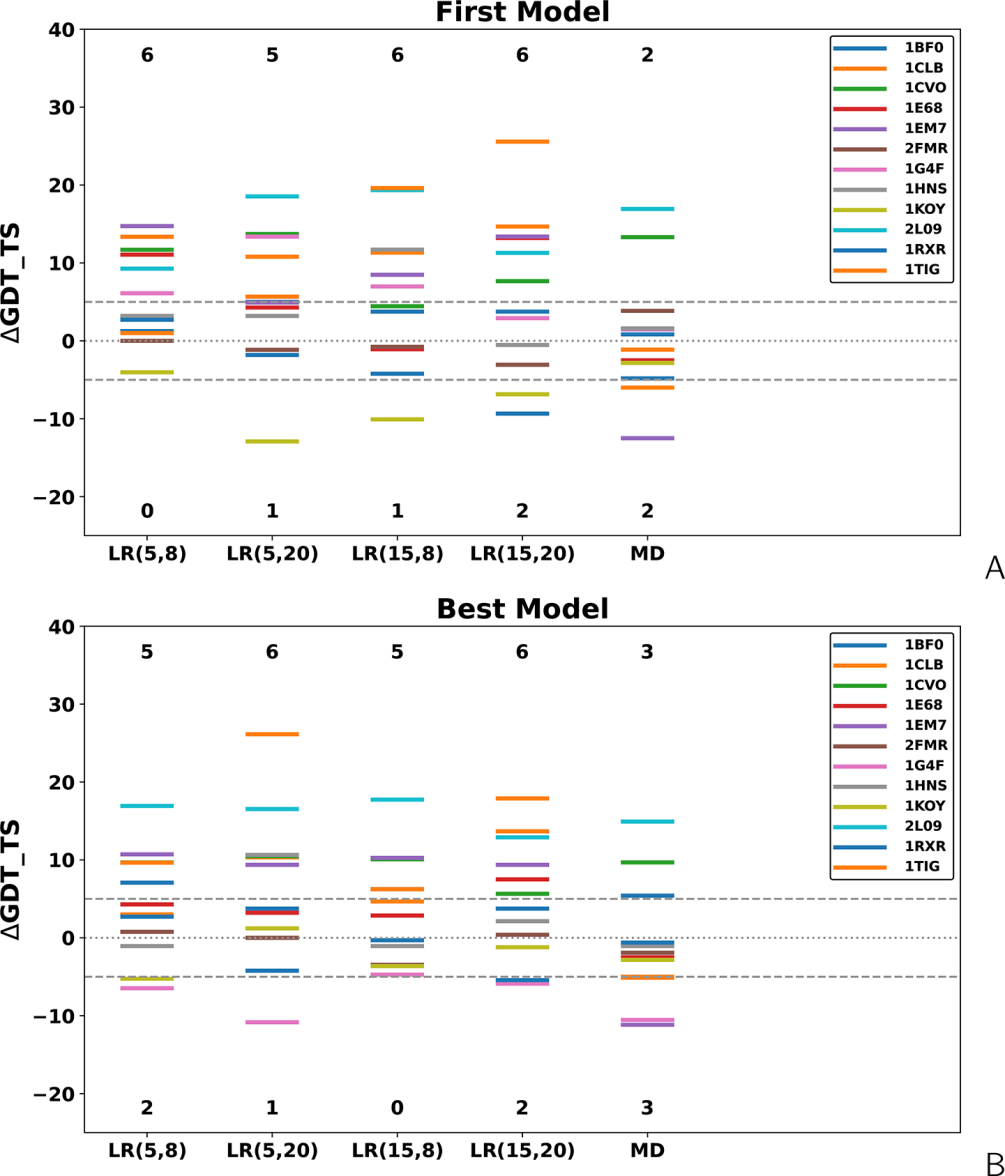
Level diagrams of the difference of the GDT_TS of the (A) first and (B) highest-GDT_TS models of the short-cross-link benchmark proteins obtained in cross-link-restrained simulations from those obtained in unrestrained simulations. *LR*(σ,*A*) denotes Lorentz-like potentials, with σ and *A* being the wall thickness and well depth, respectively (eq 8), and MD denotes MD-based potentials (eqs 3–6).

The bar plots of the Global Distance Test Total Score (GDT_TS),^59^ which is a measure of the percentage of the model that is similar to the experimental structure, for the first-choice (corresponding to the greatest probability of the respective conformational family) and the best (with the largest GDT_TS) models for the 12 proteins with synthetic SDA and DSA cross-link data (Table S4) are shown in Figure 4. The plots correspond to unrestrained simulations and simulations with the MD-determined (eqs 3–6) and with the Lorentz-like potential (eq 8). The latter were carried out with four variants of parameters, as specified in section “Cross-Link Restraints with UNRES”. The numerical values, along with the values of C^α^ RMSD and TMScore,^60^ are collected in Table S7 in the Supporting Information. In Figure 5, the level diagrams of the differences between the GDT_TS values for restrained and unrestrained simulations are shown. It can be seen from Figure 4 and Table S7 that, except for 1EM7 (an α + β protein), for which UNRES produces both the first and the best model with GDT_TS over 50, the UNRES models of the other proteins are of modest quality, with GDT_TS being from slightly over 20 to slightly over 40.

It can be seen from Figures 4 and 5 that, in all instances, the Lorentz-based potentials perform better than the MD-based potentials. Considering the changes of GDT_TS greater than ±5 units as significant (improvement or deterioration), MD-based potentials result in a remarkable improvement of the first models in two instances and deterioration also in two instances, while the Lorentz-based potentials result in remarkable model improvement in five or six instances, depending on parameters and deterioration in 0–2 instances. For the best model, the numbers of significant improvements and deteriorations increase to three for the MD-based potentials and do not change for the Lorentz-based potentials.

In our earlier work,^26^ we found that using the Lorentz-like restraints (eq 8) gave worse results, compared to using the statistical potentials (eq 7). In that work, both potentials restrained the distances between the C^α^ atoms. However, the statistical potentials were used with specific and the Lorentz-like potentials with both specific and nonspecific cross-links, many of which were incorrect. This difference could contribute to the poorer performance of the Lorentz-like restraining potentials. In this work, the Lorentz-like restraints were imposed on side-chain distances and the restraints were much more tighter than those in our previous work, resulting in much better performance. Imposing restraints on the distances between side-chain ends and not on those between the C^α^ atoms, plus the comparatively short upper distance boundary, makes it more probable that the side chains remain close to the surface of the globule.^29^

The reason for the better performance of the simple Lorentz-like potentials is likely to be their flat-bottom feature. The MD-based potentials account for the dependence of the potential of mean force of the cross-linked fragment on the cross-link geometry. However, the cross-linking reagents can very well result in the disruption of protein structure after the cross-link is formed, especially if the residues involved are closer to each other in the native structure than the length of the cross-link. In this regard, simple flat-well restraints, which mainly set the upper boundary of the distance at which the respective cross-link reagent can catch both side chains, are preferable to those with a minimum or multiple minima in the distance. Thus, the flat-bottom restraints reflect the largely qualitative nature of cross-link information. These considerations are best illustrated with the 1EM7 protein, for which five cross-links occur between the neighboring strands: Y3–K50, K4–K50, K4–T51, K10–E56, and K13–E56. With the MD-based potentials, the GDT_TS decreased from 54.02 to 41.52 for the first model and from 58.04 to 46.88 for the best model, respectively. For the Lorentz-like potential, it increased, reaching the values from 58.93 (σ = 5 Å, *A* = 20 kcal/mol) to 68.75 (σ = 5 Å, *A* = 8 kcal/mol) for the first model, and from 67.41 (σ = 15 Å, *A* = 8 or 20 kcal/mol) to 68.75 (σ = 5 Å, *A* = 8 kcal/mol) for the best model, respectively.

**Figure 6:**
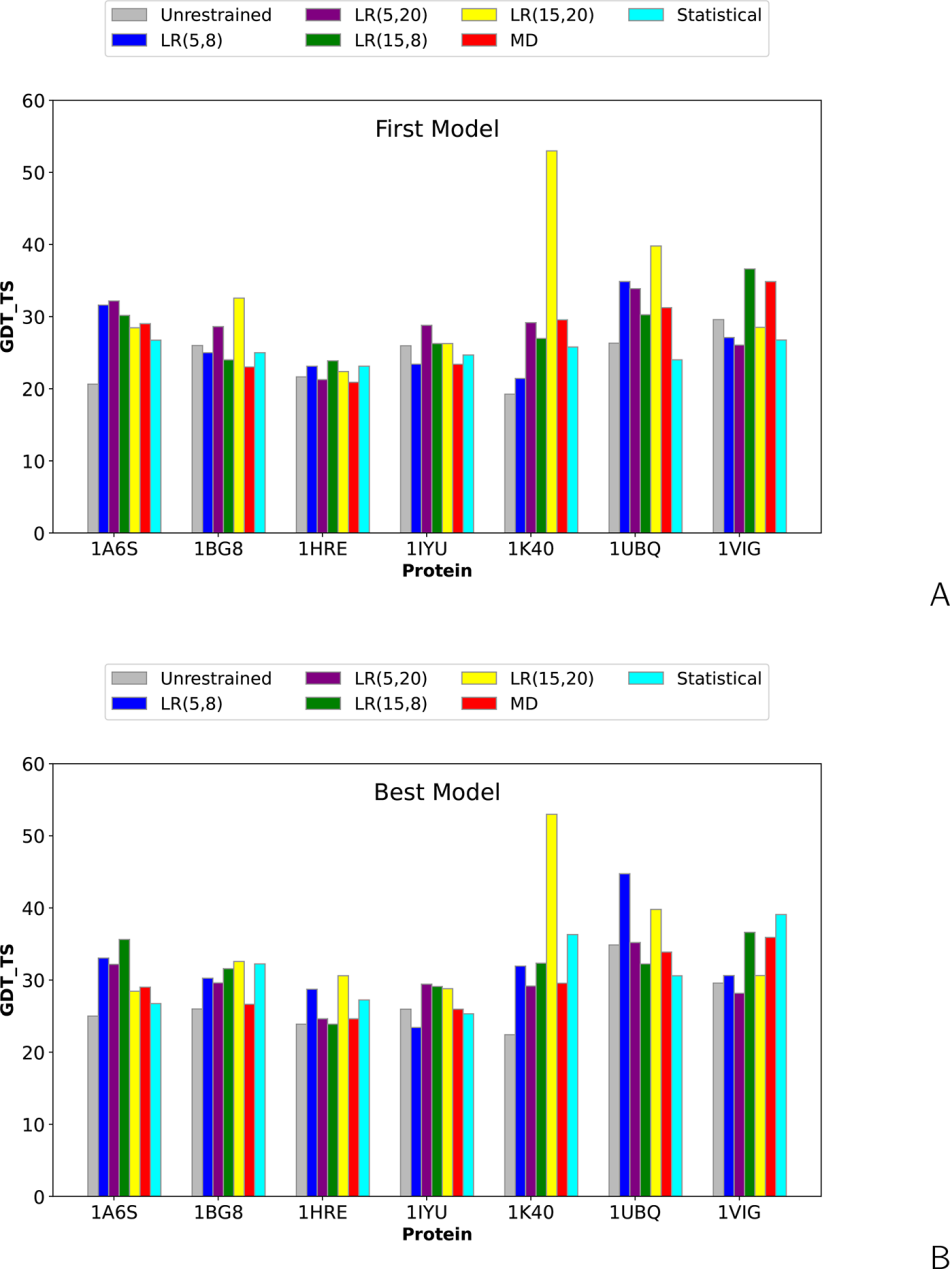
Bar plots of the global distance test total score (GDT_TS) of the (A) first and (B) highest-GDT_TS models of the BS^3^-cross-link benchmark proteins of ref (27) obtained in unrestrained UNRES simulations and cross-link-restrained simulations. *LR*(σ,*A*) denotes Lorentz-like potentials, with σ and *A* being the wall thickness and well depth, respectively (eq 8). “MD” denotes MD-based potentials (eqs 3–6), and “Statistical” denotes the statistical potentials (eq 7).

**Figure 7:**
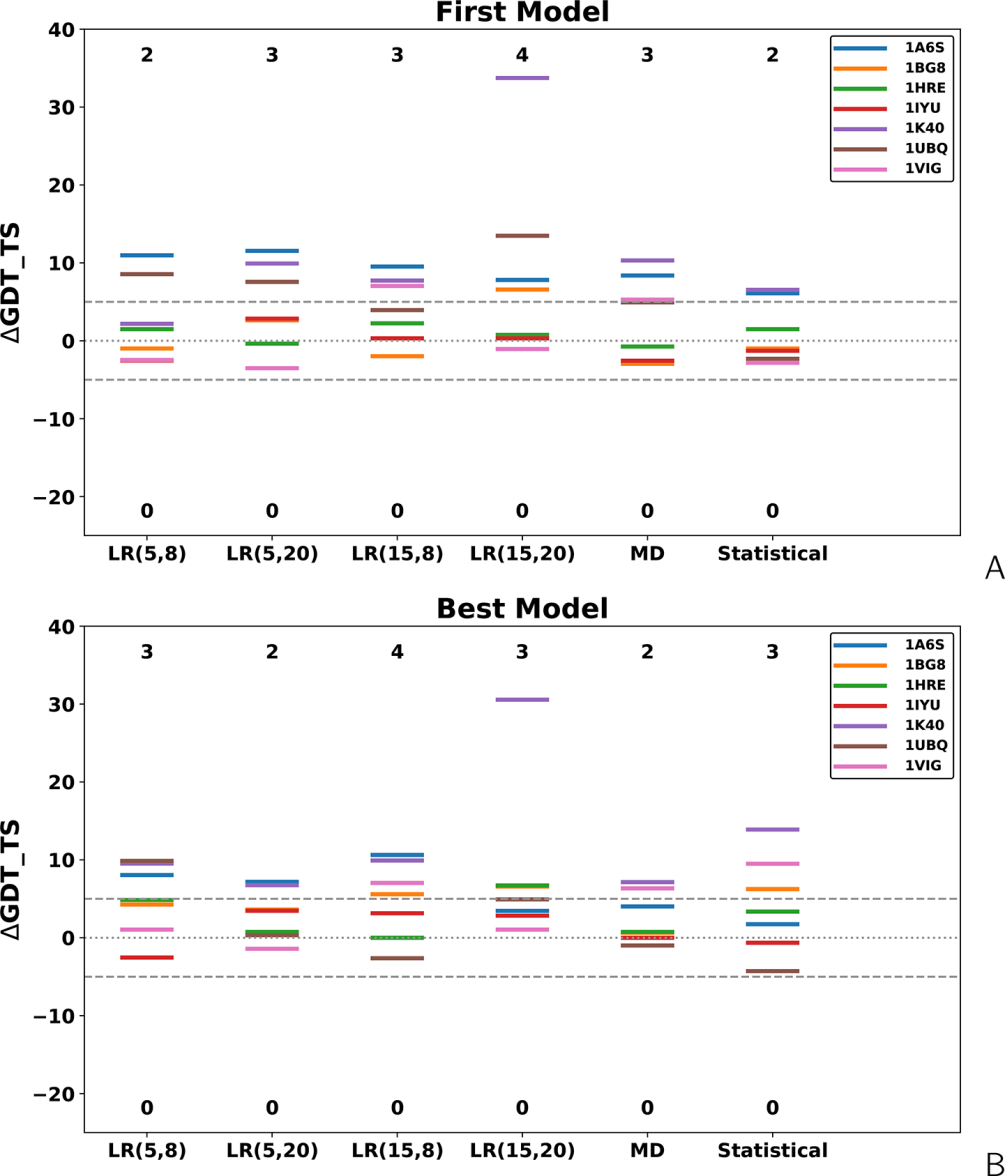
Level diagrams of the difference of the GDT_TS of the (A) first and (B) highest-GDT_TS models of the BS^3^-short-cross-link benchmark proteins of ref (27) obtained in cross-link-restrained simulations from those obtained in unrestrained simulations. *LR*(σ,*A*) denotes Lorentz-like potentials, with σ and *A* being the wall thickness and well depth, respectively (eq 8), “MD” denotes MD-based potentials (eqs 3–6), and “Statistical” denotes the statistical potentials (eq 7).

In our previous work,^27^ we evaluated the influence of the Lys-BS^3^-Lys cross-link restraints on model quality, comparing the MD-based restraining potentials with the statistical potentials. This cross-link is longer than the SDA and DSA cross-link. Therefore, we tried the Lorentz-like potentials on the 7 systems of our previous study (Table S5). Using this benchmark set also enables us to compare the results of modeling with the Lorentz-like potentials with those of the statistical potentials, because the statistical potentials are not available for the SDA-type cross-links. The GDT_TS bar plots for the first and for the best models, compared with those obtained in unrestrained calculations and the calculations with the MD-based and statistical cross-link restraints are shown in Figure 6 and the respective values are collected in Table S8 in the Supporting Information. The level diagrams depicting the differences in GDT_TS between unrestrained and restrained simulations are shown in Figure 7.

As can be seen from Figures 6 and 7 and Table S8, the Lorentz-like cross-link potentials do not result in remarkably increased numbers of significantly (over 5 GDT_TS units) improved models, compared to the MD-based or statistical potentials (2–4, depending on settings, vs 3 and 2, respectively for the first and 2 and 3, respectively, for the best models). This is a remarkable difference from the results obtained with short cross-links, in which the number of significantly improved first models obtained with the Lorentz-like potential is 2 or 3 times greater than that of the models obtained with the MD-based potential and the number of improved best models is up to 2 times greater (Figure 5). A plausible explanation of this difference is a greater length of the BS^3^ cross-links. Moreover, a closer inspection of Figures 5 and 7 indicates that the increase in GDT_TS is usually smaller for the models obtained with the longer (BS^3^) cross-link restraints, usually not exceeding 10, while a GDT_TS increase between 10 and 20 is more common with the DSA and SDA cross-link restraints. An exception is the result obtained for 1K40 with σ = 15 Å, *A* = 20 kcal/mol, for which GDT_TS increased by more than 30. On the other hand, the BS^3^ restraints produced no models significantly worse than those obtained from unassisted simulations, which suggests that restraints corresponding to longer cross-links are safer to use in modeling. This observation is consistent with the fact that the cross-link restraints could correspond to distances in distorted protein structures (by natural fluctuations or because of making another cross-link earlier). Longer and, consequently, more flexible cross-links (e.g., BS^3^) produce flatter restraint-potential wells (cf Figure 3 in ref (27)), thus compensating for the distortions.

**Figure 8:**
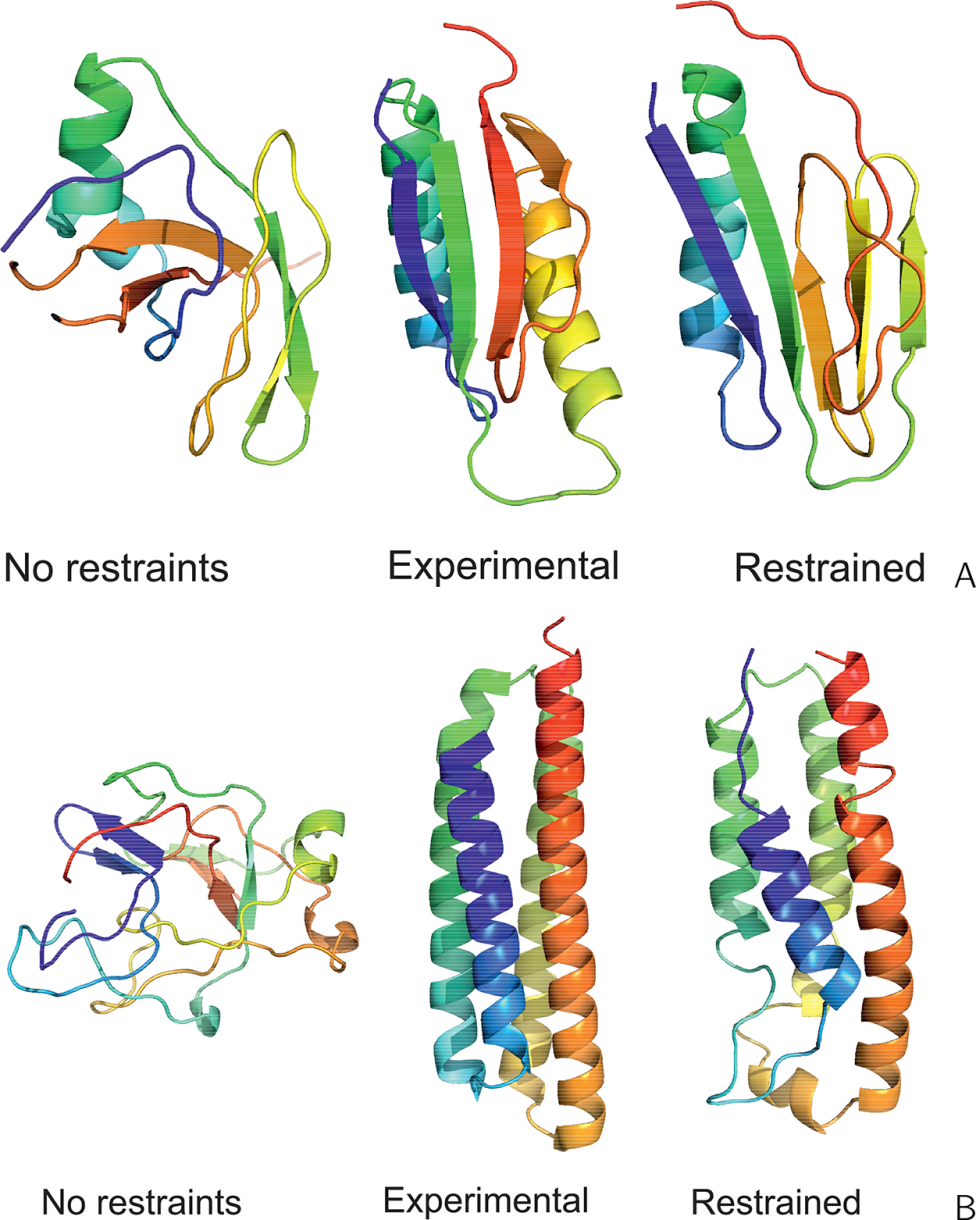
Experimental structures (center of a panel) of (A) 1TIG and (B) 1K40, compared with the respective first models of these proteins obtained in unrestrained UNRES simulations (left side of panel) and UNRES simulations restrained with the Lorentz-like cross-link potentials (right side of panel). The parameters of the Lorentz-like potentials were σ = 15 Å, *A* = 20 kcal/mol, respectively. For 1TIG (90 residues), the GDT_TS and C^α^ RMSD are 26.70 and 12.12 Å in unrestrained and 52.57 and 6.94 Å in restrained simulations, respectively. For 1K40 (126 residues), C^α^ RMSD are 19.25 and 12.12 Å in unrestrained and 52.98 and 3.92 Å in restrained simulations, respectively. The drawings were made with PyMOL.^61^

Because the cross-link restraints are usually small in number, the improvement of model quality is rather modest, mostly up to ∼20 GDT_TS units for short cross-links and up to 10 GDT_TS units for longer cross-links. This observation was also made in our earlier work.^26,27^ Nevertheless, with the Lorentz-like restraints, the improvement is significant for 1TIG (an α + β protein) of the short-cross-link benchmark set, the first model of which reached a GDT_TS value of 52.27 with σ = 15 Å, *A* = 20 kcal/mol compared to GDT_TS = 26.70 for unrestrained simulations (Table S7) and for 1K40 (an α protein) of the long-cross-link benchmark set of ref (27), for which model 1 reached GDT_TS of 52.98 with σ = 15 Å, *A* = 20 kcal/mol, compared to 19.25 with unrestrained simulations (Table S8). The experimental and simulated (without and with cross-link restraints) structures of these two proteins are shown in Figures 8A and 8B, respectively. On the other hand, the results for 1KOY (an α protein) of the short-cross-link and 1HRE (an α + β protein) of the long-cross-link benchmark set are consistently poor. Inspection of the cross-link list of those four targets (Tables S4 and S5) shows that both the number and the topological length of cross-links for 1TIG (13 cross-links, the longest cross-link closing a loop of 56 residues) and 1K40 (9 cross-links, the longest cross-link closing a loop of 99 residues) are significant, while there are only a few remarkably topologically shorter cross-links for 1KOY (3, the longest cross-link closing a loop of 33 residues) and 1HRE (6, the longest cross-link closing a loop of 19 residues).

**Figure 9:**
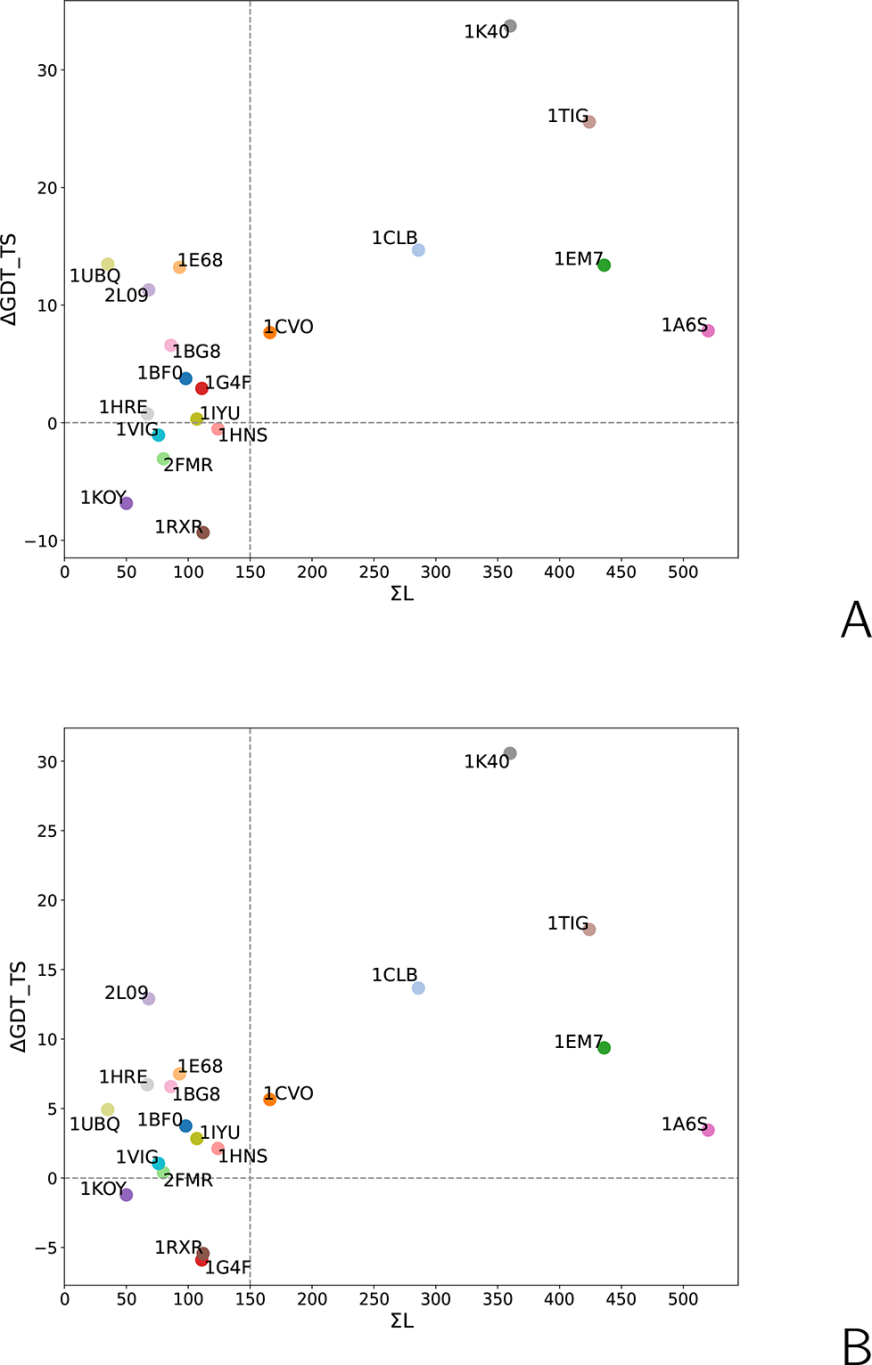
Relationship between the sum of cross-link topological lengths (Σ*L*) with the difference between the GDT_TS of models obtained with Lorentz-type cross-link restraints (eq 8) with σ = 15 Å and *A* = 20 kcal/mol and those obtained in unrestrained simulations (Δ*G*DT_TS) for the (A) first and (B) highest-GDT_TS models of the 19 benchmark proteins with synthetic cross-link restraints.

The above observation suggests that the increase of GDT_TS of the structures obtained from cross-link-assisted modeling could be related to the number of cross-links and their topological lengths. In Figures 9A and 9B, the differences in the GDT_TS values between the first and best models, respectively, of the structures obtained with cross-link restraints and those from unrestrained simulations (ΔGDT_TS) are plotted against the sum of the topological lengths of all cross-links (Σ*L*) defined by eq 10:
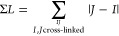
10It can be seen that Δ*G*DT_TS is correlated with Σ*L*. The correlation is weak; however, for Σ*L* > 150, ΔGDT_TS is always positive, in most cases, exceeding 5 units. For small Σ*L*, a substantial increase in GDT_TS can also be obtained if the few cross-links happen to correspond to contacts that define the fold topology. This occurs for 1UBQ, (three cross-links, two long-range only), 2L09 (three long-range cross-links), and 1E68 (four cross-links, four long-range) for which GDT_TS increased by 10 units or more (Figure 9). On the other hand, low Σ*L* more often results in small or no improvement or even deterioration of model quality.

In Figure S8 in the Supporting Information, the correlation diagrams of ΔGDT_TS with the number of cross-links (*N*_XL_), the maximum cross-link length (*L*_max_), and Σ*L* are shown for all variants of the Lorentz-like restraint function and for the MD-derived restraints, for both the first and the best models of the 19 benchmark proteins with synthetic cross-link data. It can be seen that if any of these measures exceeds a certain threshold, ΔGDT_TS is remarkably positive. Of those, Σ*L* > 150 consistently points to the greatest number of targets with positive ΔGDT_TS and can, therefore, be considered a descriptor that predicts the capacity of a given set of cross-link restraints to improve model quality. It combines the number of cross-links with their topological distances. Defining long-range residue–residue contacts is very important, because the errors inherent in a force field accumulate with increasing segment length and, consequently, long-range contacts are less likely to be reproduced correctly in modeled structures. On the other hand, a greater number of restraints corrects force-field errors in a larger number of segments.

The Σ*L* descriptor does not fully define the capacity of a cross-link set to improve modeled-structure quality. As can be seen from Figure 9, the ΔGDT_TS of the six proteins with Σ*L* > 150 does not exactly follow the increase of Σ*L*. The maximum ΔGDT_TS occurs for 1K40, which has a moderate Σ*L*, while that for 1A6S, which has the largest Σ*L* and the greatest number of cross-links (20; see Table S5), is below 5. The exceptional model improvement for 1K40 probably results from its simple four-helix-bundle topology (Figure 8B). The model-improvement capacity of a cross-link set probably depends on whether the cross-link restrains are imposed on the distances between the residues in regions that the force field does not handle well. However, if the experimental structure is unknown, there is no way to determine these regions. Therefore, a crude assessment the model-improvement capacity of a cross-link set based on Σ*L* threshold seems to be a sensible solution. Also note that the threshold of 150 has been established based on the benchmark set of small proteins used in our study and could change if the set is extended, especially by larger proteins. Moreover, because of the small size of the protein-benchmark set used in this study, we refrained from using multiple descriptors to determine cross-link-set capacity to improve the quality of modeled structures.

### Experimental Cross-Link Data

**Figure 10:**
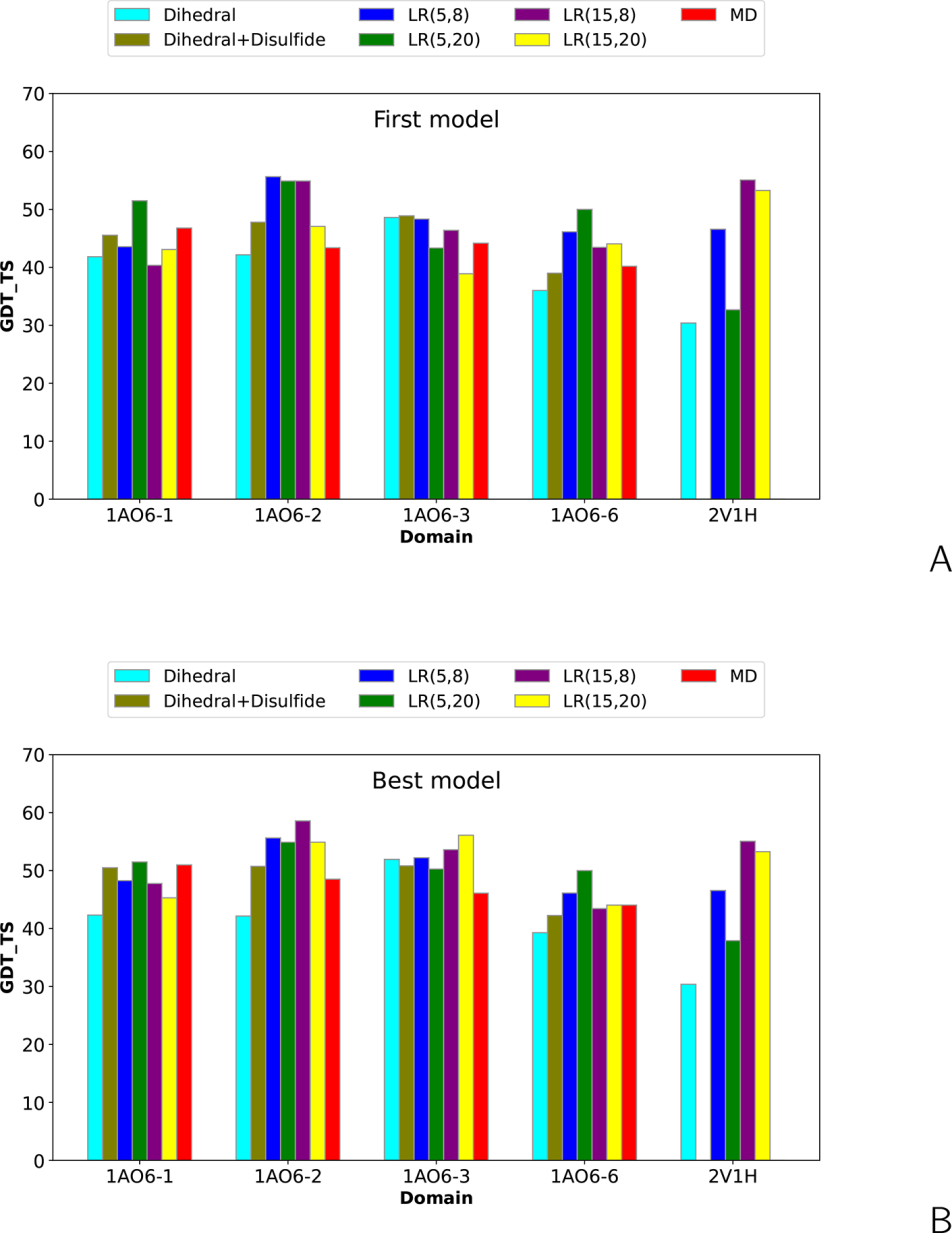
Bar plots of the global distance test total score (GDT_TS) of the (A) first and (B) highest-GDT_TS models of repeats 1, 2, 3, and 6 of human serum albumin (PDB: 1AO6) and horse myoglobin (PDB: 2V1H) obtained in unrestrained UNRES simulations and cross-link-restrained simulations. *LR*(σ,*A*) denotes Lorentz-like potentials, with σ and *A* being the wall thickness and well depth, respectively (eq 8), and “MD” denotes MD-based potentials (eqs 3–6).

The MD-based cross-link-restraint potentials were available only for the four selected repeats of human serum albumin (AO1-1, AO1-2, AO1-3, and AO1-6). Therefore, for these systems, we carried out the simulations with both the MD-based and Lorentz-like restraints. The MD-based potentials are not available for most of the cross-links used in the experiments on horse myoglobin (2V1H) reported in ref (8). Therefore, we used only the Lorentz-like potentials for this target. The cross-links are summarized in Table S5. The bar plots of the GDT_TS for the first and highest GDT_TS models are shown in Figure 10, and the numerical data are collected in Table S8 in the Supporting Information.

**Figure 11:**
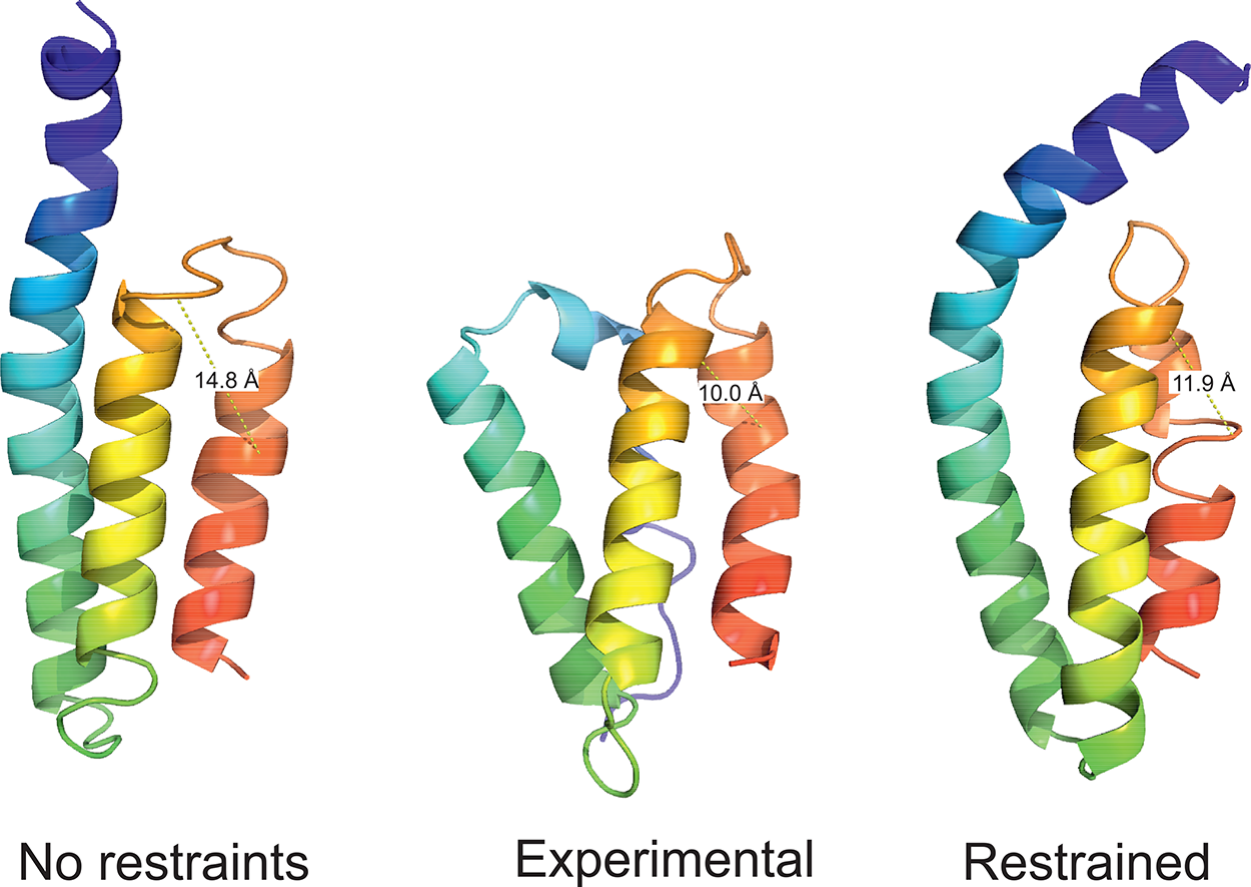
Experimental structure (center of the panel) of the sixth repeat of human serum albumin (1AO6-6, 84 residues) compared with the first model of this protein obtained in unrestrained UNRES simulations (left side of the panel) and UNRES simulations restrained with the Lorentz-like cross-link potentials (right side of the panel). The parameters of the Lorentz-like potentials were σ = 5 Å, *A* = 20 kcal/mol, respectively. The GDT_TS and C^α^ RMSD are 38.10 and 10.35 Å in unrestrained and 46.63 and 10.66 Å in restrained simulations, respectively. The only nonlocal cross-link and the respective side-chain-end distances are shown in all panels. The drawings were made with PyMOL.^61^

We analyze the results for repeats 1, 2, 4, and 6 of serum albumin first. For all these systems, Σ*L* < 150 (not counting the natural S–S links; see Table S5). Of all repeats, AO6-2 has the biggest Σ*L* = 138. It can be seen that, for this repeat, a major GDT_TS increase was obtained in all simulations with the Lorentz-like potential except for the first model resulting from the simulations with σ = 15 Å and *A* = 20 kcal/mol. It can also be noted that a remarkable GDT_TS increase resulting from modeling with the Lorentz-like cross-link-restraining potentials is observed consistently for the AO6-6 repeat even though Σ*L* = 22 only (Table S5). For this repeat, the GDT_TS values obtained in unassisted modeling (with or without natural disulfide links) are significantly lower than those for the other repeats (see Table S9 and Figure 10). The K557–K571 cross-link that bridges the two α-helical segments results in a significant improvement of model quality (Figure 11). A similar situation occurred in our participation in the CASP10 experiments within the WeFold initiative,^62^ in which a couple of well-predicted distance restraints for the T0740 target resulted in the best prediction of the structure of this target.

It can be seen from Figure 10 and Table S5 that, consistent with the results presented in the section entitled “Synthetic Cross-Link Data”, using the MD-derived restraint potentials results in only incremental GDT_TS increases at best and in small decreases in GDT_TS in most of the calculations.

Note that, in ref (4), higher-quality structures of 1AO6 domains were obtained. However, the structures were modeled with ROSETTA^16^ and the information from contact prediction was used, while we applied disulfide-bridge and cross-link restraints exclusively.

**Figure 12:**
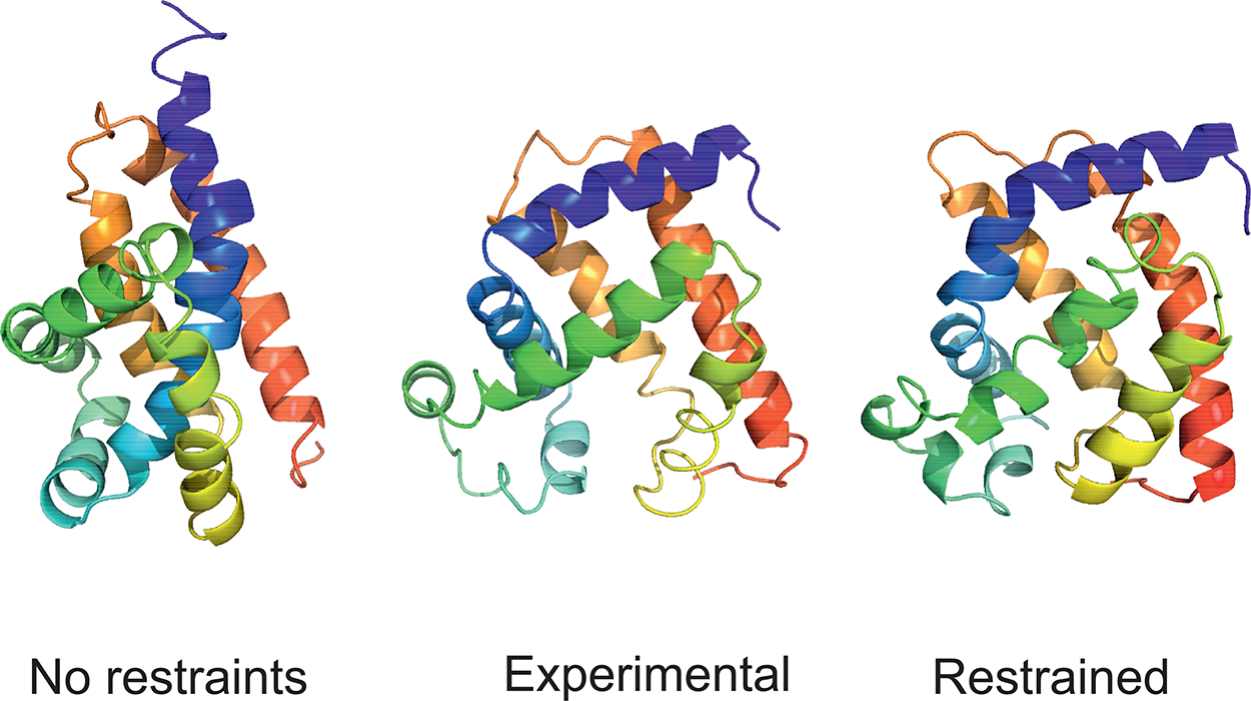
Experimental structures (center of the panel) of horse myoglobin (2V1H, 153 residues) compared with the first model of this protein obtained in unrestrained UNRES simulations (left side of the panel) and UNRES simulations restrained with the Lorentz-like cross-link potentials (right side of the panel). The parameters of the Lorentz-like potentials were σ = 15 Å, *A* = 8 kcal/mol, respectively. The GDT_TS and C^α^ RMSD are 30.39 and 9.23 Å in unrestrained and 55.07 and 3.90 Å in restrained simulations, respectively. The drawings were made with PyMOL.^61^

The last system, horse myoglobin (PDB: 2V1H) also is an all-α-helical protein. The Σ*L* value is 1367. As can be seen from Table S5, the cross-links correspond to a significant number of long-range contacts thus helping to pack the segments correctly. Without the cross-link restraints, model 1 (which also is the best model) has a low GDT_TS (30.39) and an high C^α^-RMSD value (9.2 Å). With the Lorentz-like cross-link restraints, major model improvement is obtained in most calculations, with the best results corresponding to σ = 15 Å and *A* = 8 kcal/mol. The GDT_TS for the first and best models increased to 55.07 and C^α^ RMSD dropped to 3.9 Å. This result is better than that obtained by modeling with MEDUSA,^12^ which is an all-atom approach, reported in ref (8), in which RMSD was ∼5 Å. The resulting structure, along with the experimental 2V1H structure and the structure obtained without cross-link restraints is shown in Figure 12. Inspection of the respective bar plot in Figure 10 demonstrates that the model quality obtained in cross-link-assisted simulations using the Lorentz-like potential with σ = 5 Å is remarkably worse than that obtained with σ = 15 Å. Additionally, if the small σ is combined with deeper potential well (*A* = 20 kcal/mol), the model quality is not much greater than that obtained without restraints. This feature seems to be caused by the presence of a substantial fraction of false cross-link restraints (Table S6). When σ is greater, the false restraints are not strictly enforced and, consequently, better models are obtained. A more shallow restraint-potential well also contributes to reducing the effect of false restraints. In our earlier work,^43^ we argued that false distance restraints are largely contradictory and, therefore, it is enough to use the bounded Lorentz-like function to effectively eliminate them provided that the number of restraints is sufficiently large. However, when the number of restraints is small (as for cross-link restraints), false restraints can be satisfied along with true restraints resulting in poorer-quality models.

## Conclusions

In this work, we evaluated the effect of cross-link restraints, imposed on the side chains of cross-linked residues or cross-linked termini, on the quality of models of protein structures obtained by extensive conformational search with the coarse-grained UNRES model, by using the protocol based on MREMD simulations developed in our earlier work.^41^ We considered the short cross-links formed by three heterobifunctional cross-linking reagents, namely, azido benzoic acid succinimide (ABAS), triazidotriazine (TATA), and succinimidyldiazirine (SDA), and two homobifunctional reagents (namely, disuccinimidyl adipate (DSA) and disuccinimidyl glutarate (DSG)). Two types of cross-links potentials were considered. Those of the first type are based on analytical expressions fitted to the potentials of mean force of the respective cross-linked fragments determined by all-atom MD simulations of model systems and depend on side chain distance and orientation (eqs 3–6), while those of the second type have the form of a simple Lorentz-like flat-bottom potential (eq 8), which has an upper boundary. Of the heterobifunctional cross-linking reagents, the binding modes are known only for SDA and, consequently, we determined the MD-based potentials only for the SDA and DSA cross-links; those for DSG were determined in our earlier work.^27^ Additionally, we also compared the performance of the simple Lorentz-like cross-link restraining potentials corresponding to a longer suberic-acid (BS^3^, a homobifunctional reagent) cross-link with that of MD-based and statistical potentials reported in our previous work.^27^

For the systems with synthetic cross-link data (a total of 12 small proteins plus 7 additional small proteins studied in our previous work^27^) and those with experimental cross-link data (a total of four systems), the simple Lorentz-like potentials turned out to produce models more similar to the experimental structures (with higher GDT_TS and lower C^α^-RMSD values) than the more-sophisticated MD-based potentials. The reason for this seems to be that the latter have minima at the side-chain–side-chain distances longer than the side-chain–side-chain contact distances in the native structures. For the longer BS^3^-type cross-links, the results obtained with the two kinds of potentials were more similar, most likely because of the greater flexibility of the longer cross-links, which is manifested as a more flat MD-based cross-link-distance potential (Figure 3 in ref (27)). Conversely, a simple flat-bottom potential with an upper distance boundary corresponding to the respective cross-link length will not force two cross-linked residues to assume a distance too long to produce a good model. This observation also conforms with the character of the cross-link experiments, in which the pairs of residues whose side chains are located on the surface and are closer to each other than the cross-linking-reagent dimension are picked. Note that the structure can be largely distorted or even disrupted after a cross-link is formed. Thus, the MD-based potentials produce models of hypothetical structures, which would be obtained after the cross-linkable residues are cross-linked rather than those of unperturbed native structures.

The modeling experiments with both synthetic and experimental cross-link data carried out in this and in our previous work^27^ demonstrated that the improvement of model quality depends on the number of cross-links and their topological lengths (the number of residues in the loop closed by a cross-link). If many long-range cross-links are present, GDT_TS can increase even by more than 30 units, as observed for the 1K40 protein (see Figure 6, as well as Table S8). A quantitative measure of the number of long-range cross-links is the sum of topological cross-link lengths, Σ*L* (eq 10); Figure 9 shows that, when this quantity exceeds 150, GDT_TS increases significantly. Therefore, when planning cross-linking experiments for a given system, the cross-linking reagents should be selected to provide the greatest Σ*L*. The heterobifunctional cross-linking reagents seem to be more appropriate than the homobifunctional ones, because they can link more combinations of residue pairs. On the other hand, a smaller Σ*L* does not necessarily imply poor model quality, because the scarce cross-link restraints can be essential in correcting force-field inaccuracy, as demonstrated with the examples of 1UBQ, 2L09, and 1E68, for which GDT_TS increased by 10 units or more, despite low Σ*L* values (see Figure 9).

The example of horse myoglobin (Figure 10, as well as Table S6) demonstrates that sufficient cross-link information can result in major model-quality improvement, even with a substantial number of “false” cross-links between spatially distant residues. In such a case, it seems that looser Lorentz-like restraints with a greater wall thickness (σ) and a shallower potential well (*A*) in eq 8 should be applied to reduce the effect of false restraints. On the other hand, the presence of contradictory restraints can very well indicate significant conformational mobility, suggesting that time- or replica-averaged cross-link restraints should be used in modeling. This remark particularly applies to intrinsically disordered proteins (IDPs) and proteins with intrinsically disordered regions (IDRs). Research on implementing averaged cross-link restraints to determine the conformational ensembles of IDPs/IDRs, as well as the conformational mobility of proteins, is now being carried out in our laboratory.

## Data Availability

The UNRES software with cross-link-assisted-modeling capacity is available at https://unres.pl/downloads and https://projects.task.gda.pl/eurohpcpl-public/unres, under the GPL v3 license. The numerical values of GDT_TS shown in Figures 4–7, 9, and 10 are collected in Tables S7–S9 of the Supporting Information.

## Supporting Information Available

Atom charges of model compounds pertaining to the SDA cross-links (Figure S1); plots of the MD-based cross-link potentials (Figures S2–S7) and parameters of these potentials (Tables S1–S3); relationships between various measures of the number and topological length of cross-links and GDT_TS difference of the first (Figure S8) and the best models (Figure S9); synthetic (Tables S4 and S5) and experimental (Table S6) cross-link information; results of modeling for the benchmark proteins with synthetic (Tables S7 and S8) and experimental (Table S9) cross-link restraints (PDF)
Archive file with data in machine-readable format, including input and parameter files, as well as the PDB files of the structures discussed in the paper (ZIP)
