# Supplementary material for: Assessment of Two Restraint Potentials for Coarse-Grained Chemical-Cross-Link-Assisted Modeling of Protein Structures
Source: J Chem Inf Model. 2024 Feb 12;64(4):1377–93. doi: 10.1021/acs.jcim.3c01890 (PMC10900291; doi:10.1021/acs.jcim.3c01890)
Supplement: Supplementary file 1 — ci3c01890_si_001.pdf [file ci3c01890_si_001.pdf]

Supporting Information
Assessment of two restraint potentials for coarse-grained chemical-cross-link-assisted modeling of protein structures

Mateusz Leśniewski,[†] Maciej Pyrka,[†,‡] Cezary Czaplewski,[†] Nguyen Truong Co,[†] Yida Jiang,[¶] Zhou Gong,[§] Chun Tang,[¶] and Adam Liwo[*,†]

†*Faculty of Chemistry, University of Gdańsk, Fahrenheit Union of Universities, ul. Wita Stwosza 63, 80-308 Gdańsk, Poland*

‡*Department of Physics and Biophysics, University of Warmia and Mazury, ul. Oczapowskiego 4, 10-719 Olsztyn, Poland*

¶*College of Chemistry and Molecular Engineering & Center for Quantitative Biology & PKU-Tsinghua Center for Life Sciences & Beijing National Laboratory for Molecular Sciences, Peking University, Beijing 100871 China*

§*Innovation Academy of Precision Measurement Science and Technology, Chinese Academy of Sciences, 30 W. Xiao Hong Shan, Wuhan 430071 China*

E-mail: adam.liwo@ug.edu.pl

Phone: +48 58 5235124. Fax: +48 58 5235012

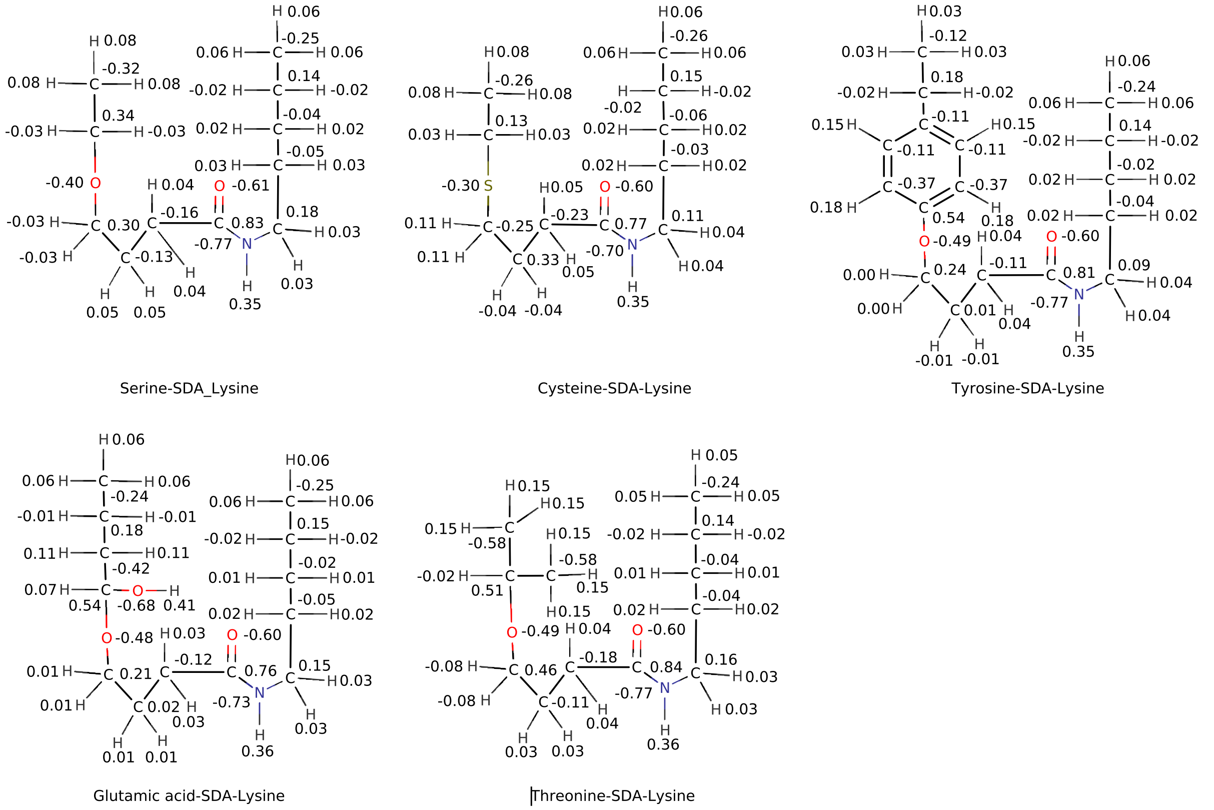

Figure S1: Charges on the atoms (electron charge units) of the model compounds selected for the determination of MD-based X-SDA-Lys potentials of mean force by all-atom MD simulations. The charges were determined by fitting to the molecular electrostatic potential calculated by the RHF 6-31G* *ab initio* method.

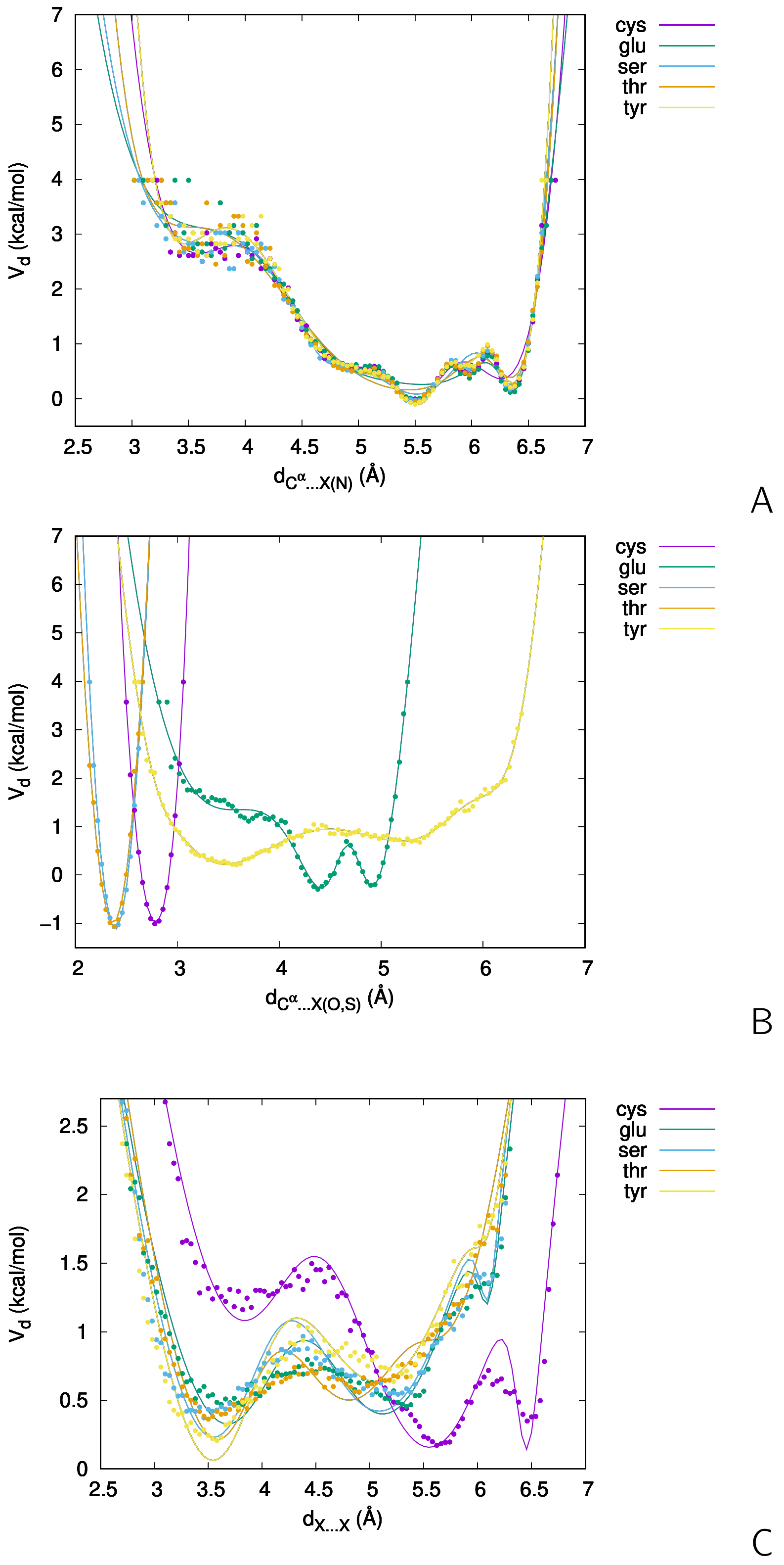

Figure S2: Potentials of mean force (filled circles) and fitted analytical expressions (lines; eq 4 of the main text) of the $C^{\alpha}\cdots X(N)$ (A) and $X(O)\cdots C^{\alpha}$ (B) and $X(N)\cdots X(O)$ (C) virtual-bond lengths for the Z-SDA-Lys cross-link systems, where Z denotes the aminoacid residue that binds to the photoactive site of SDA, X(O) denotes the photoactive site, and X(N) denotes the lysine-binding site of the SDA.

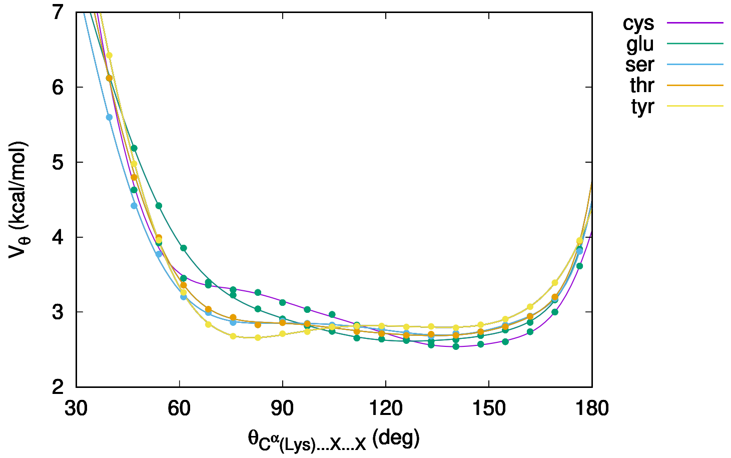

A

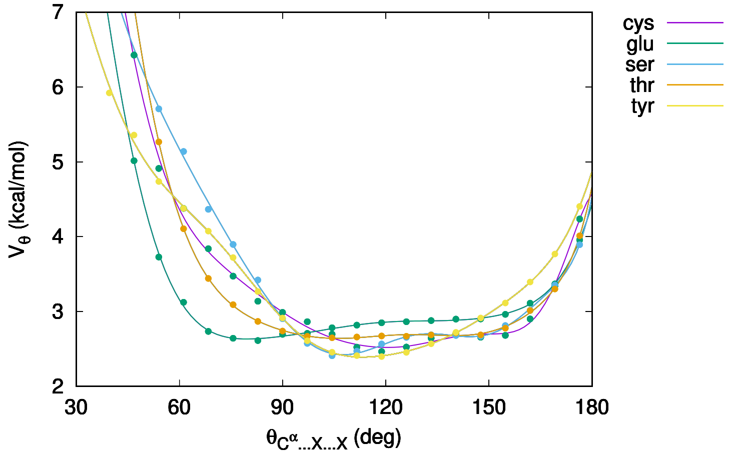

B

Figure S3: Potentials of mean force (filled circles) and fitted analytical expressions (lines; eq 5 of the main text) of the $C^\alpha \cdots X(N) \cdots X(O)$ (A) and $X(N) \cdots X(O) \cdots C^\alpha$ (B) virtual-bond angles for the Z-SDA-Lys cross-link systems, where Z denotes the aminoacid residue that binds to the photoactive site of SDA, X(O) denotes the photoactive site, and X(N) denotes the lysine-binding site of the SDA.

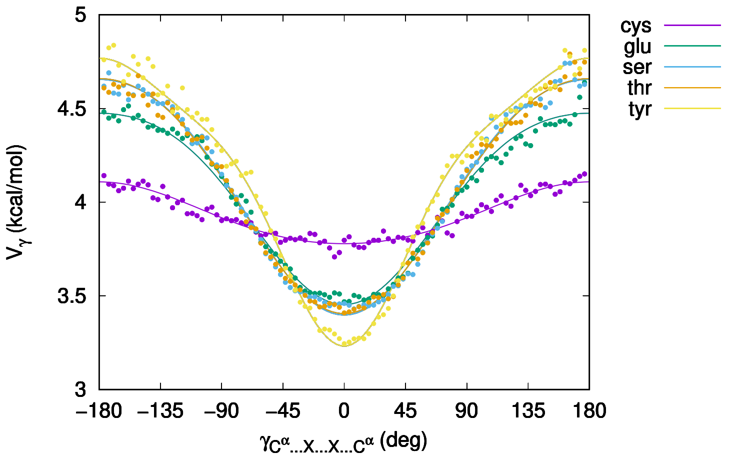

Figure S4: Potentials of mean force (filled circles) and fitted analytical expressions (lines; eq 6 of the main text) of the $C^{\alpha} \cdots X(N) \cdots X(O) \cdots C^{\alpha}$ virtual-bond-dihedral angles averaged over the adjacent virtual-bond-angles θ for the Z-SDA-Lys cross-link systems, where Z denotes the aminoacid residue that binds to the photoactive site of SDA, X(O) denotes the photoactive site, and X(N) denotes the lysine-binding site of the SDA.

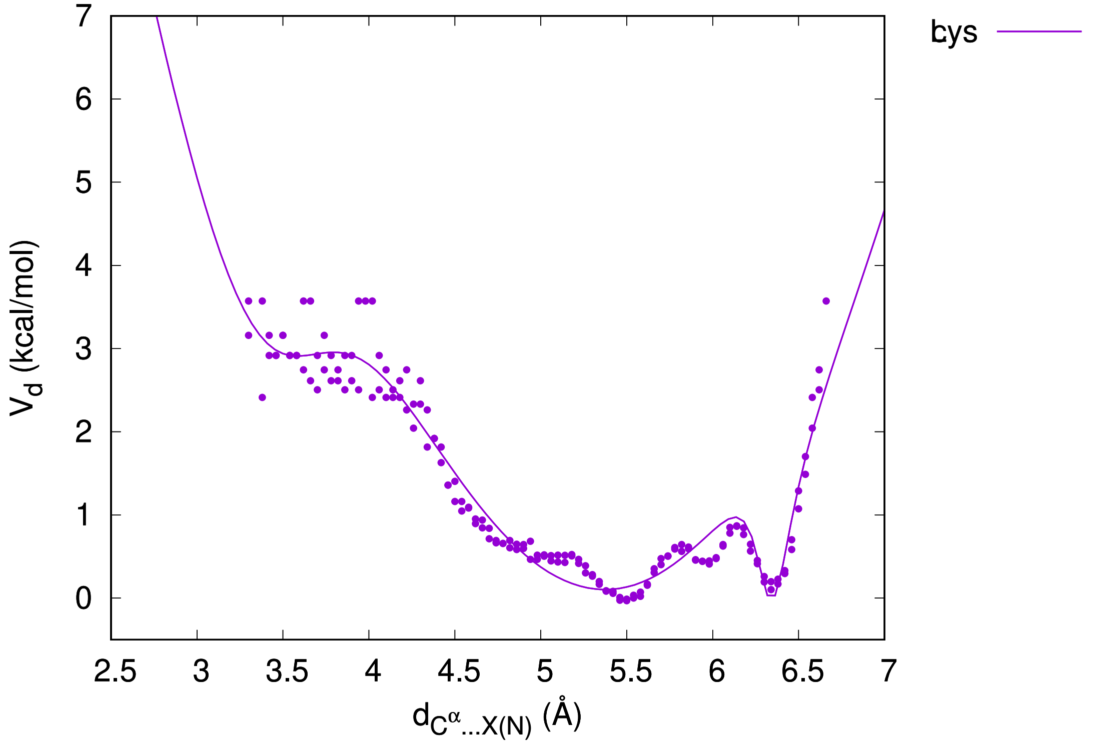

A

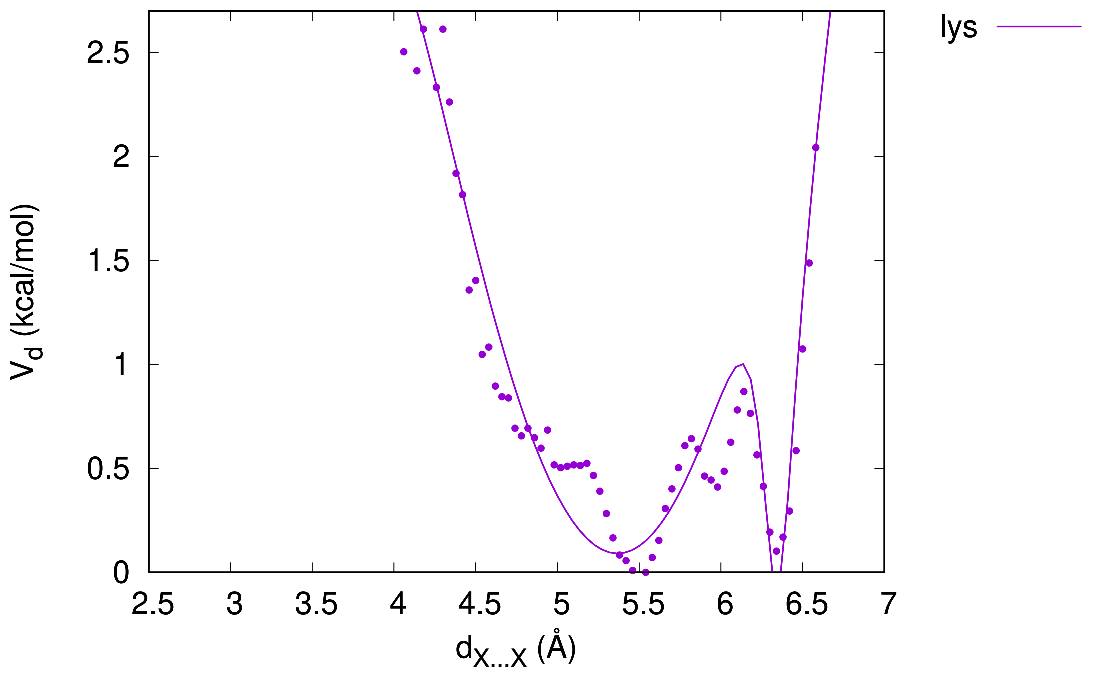

A

Figure S5: Potentials of mean force (filled circles) and fitted analytical expressions (lines; eq 4 of the main text) of the $C^{\alpha} \cdots X$ (A) virtual-bond and $X \cdots X$ (B) virtual-bond lengths for the Lys-DSA-Lys cross-link system, where X denotes a lysine-binding site of DSA.

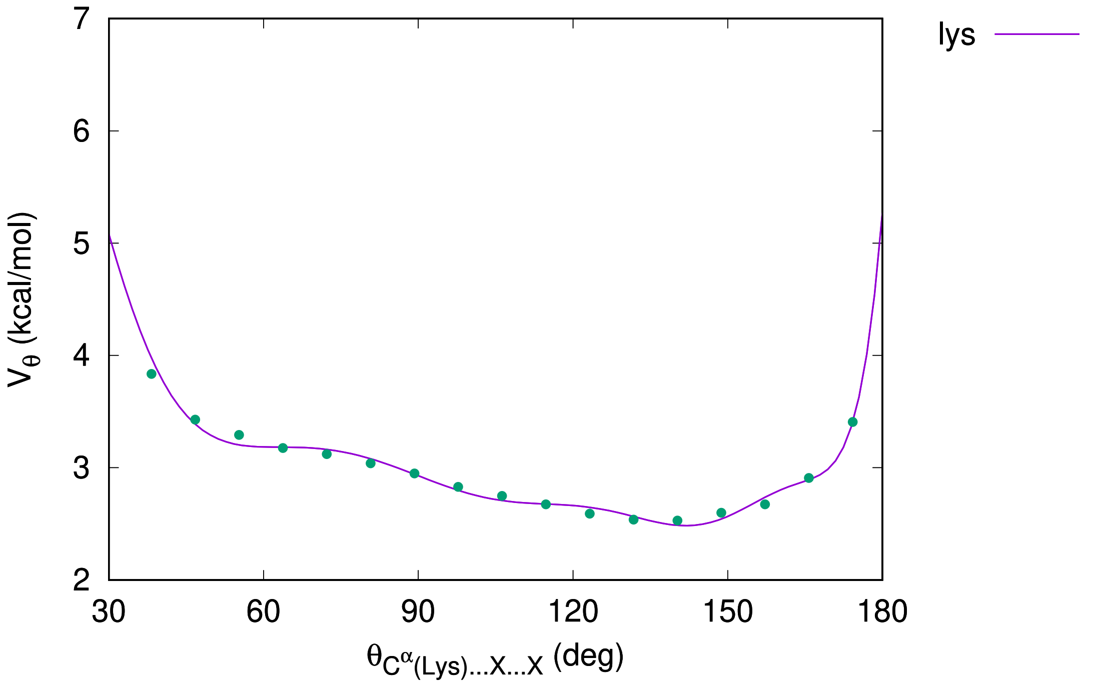

Figure S6: Potential of mean force (filled circles) and fitted analytical expressions (lines; eq 5 of the main text) of the $C^\alpha \cdots X \cdots X$ and $X \cdots X \cdots C^\alpha$ virtual-bond angles for the Lys-DSA-Lys cross-link system, where X denotes the lysine-binding site of DSA.

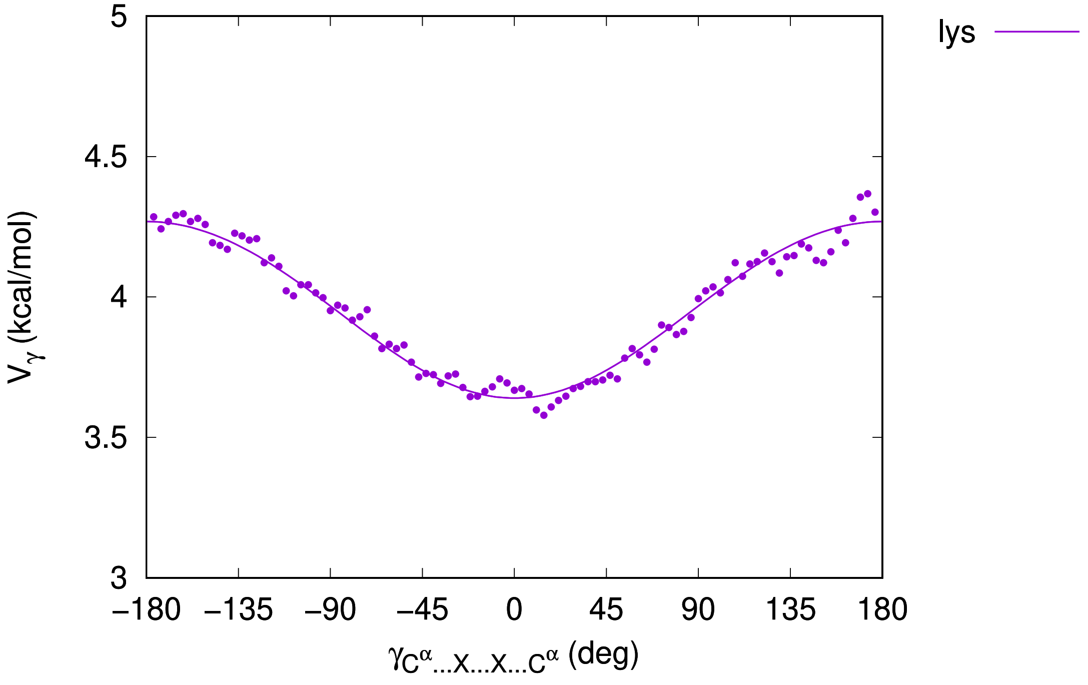

Figure S7: Potential of mean force (filled circles) and the fitted analytical expression (line; eq 6 of the main text) of the $C^\alpha \cdots X \cdots X \cdots C^\alpha$ virtual-bond-dihedral angles averaged over the adjacent virtual-bond-angles θ for the Lys-DSA-Lys cross-link system.

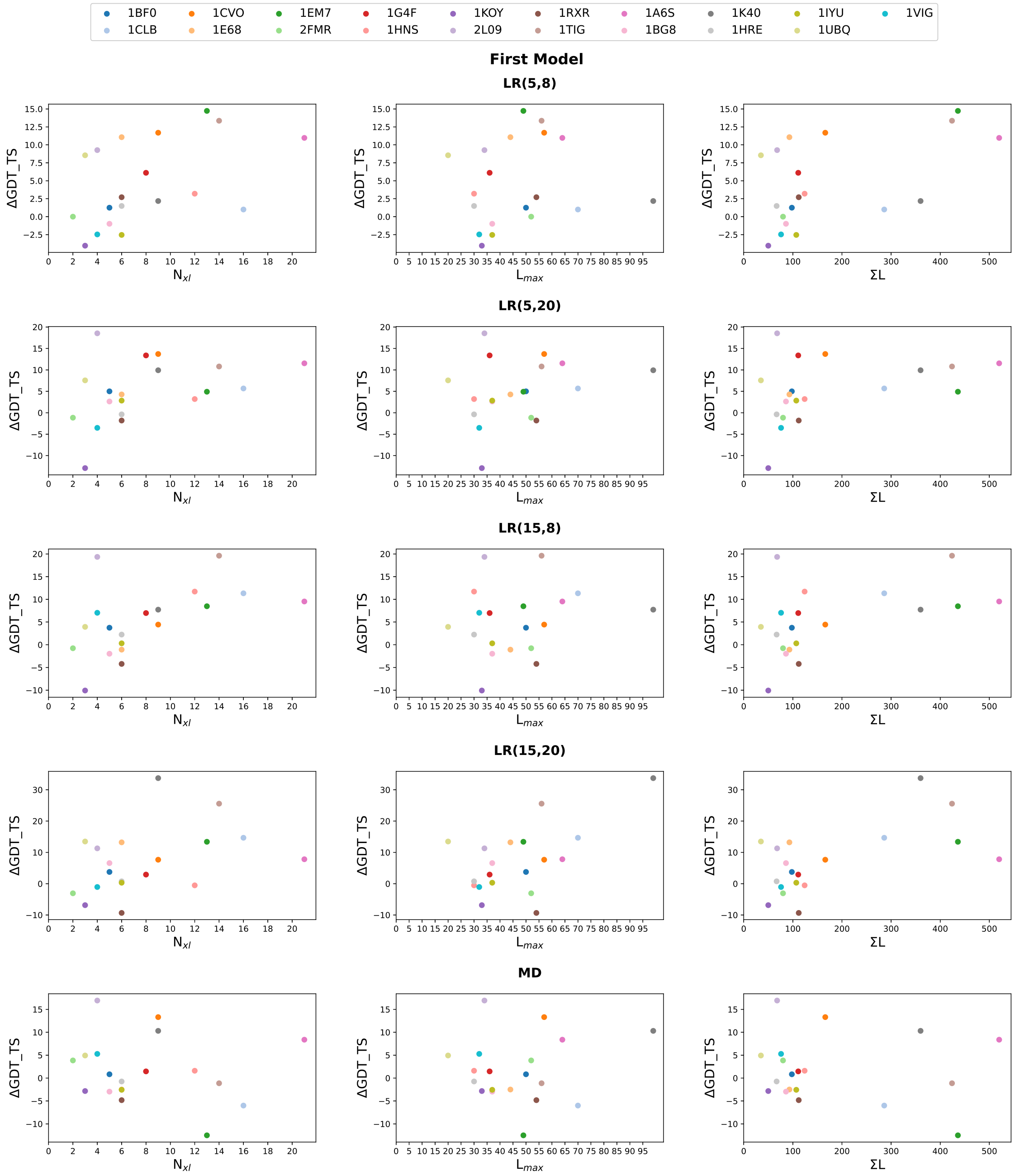

Figure S8: Relationship between the number of cross links (N_{Xl}, left panels), the topological length of the longest cross-link (L_{max}, defined as the number of residues in the loop bridged by a cross-link, middle panels) and the sum of cross-link topological lengths (ΣL, right panels) and the difference between the GDT_TS of models obtained with Lorentz-type cross-link restraints (eq 8 of the main text) for different restraint types (indicated in the respective panels) and the first models obtained from simulations. LR(σ,A) denotes Lorentz-like potentials, with σ and A being the wall thickness and well depth, respectively (eq 8) of the main text, and MD denotes MD-based potentials (eqs 3 – 6) of the main text.

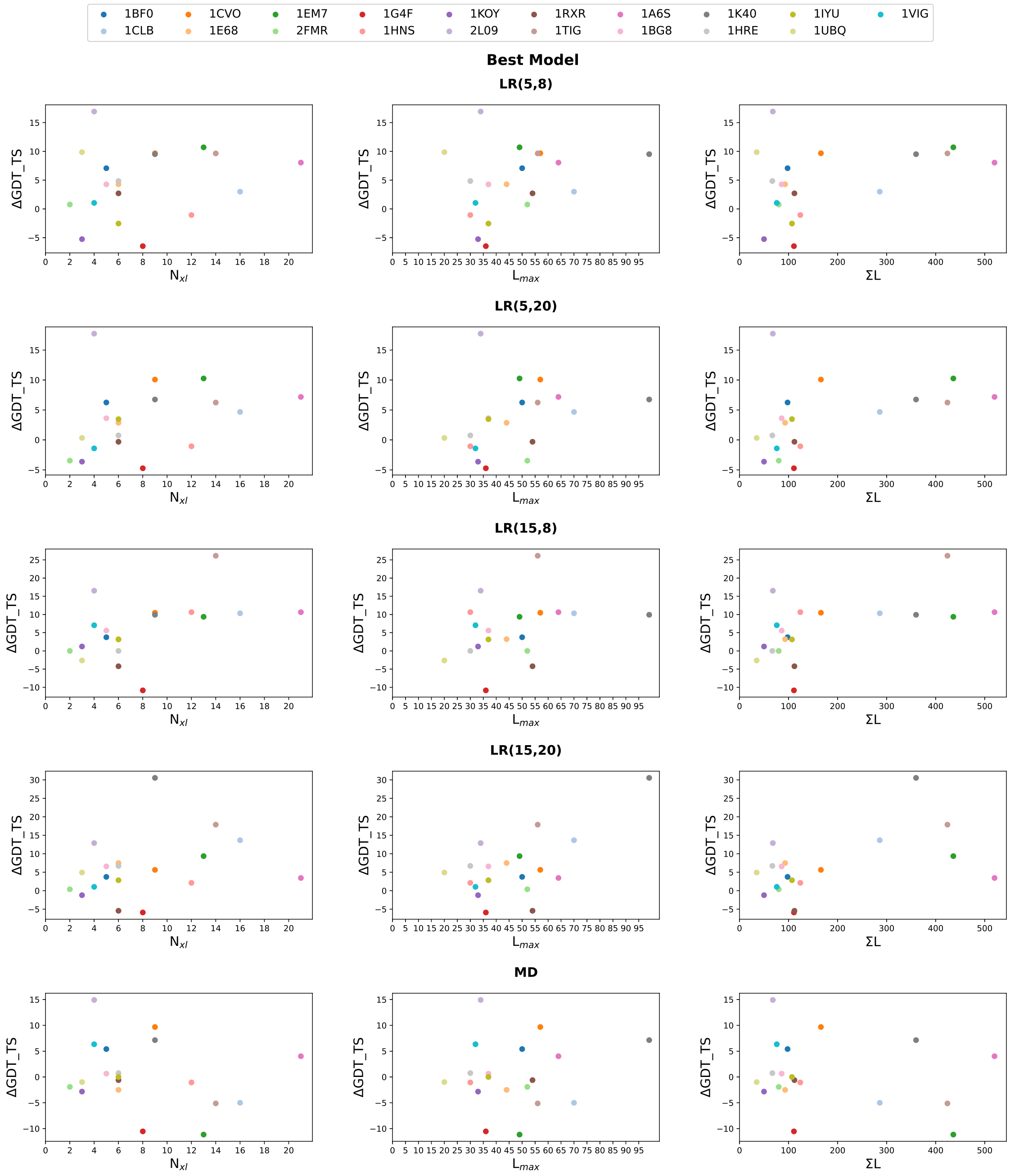

Figure S9: Relationship between the number of cross links (N_{Xl}, left panels), the topological length of the longest cross-link (L_{max}, defined as the number of residues in the loop bridged by a cross-link, middle panels) and the sum of cross-link topological lengths (ΣL, right panels) and the difference between the GDT_TS of models obtained with Lorentz-type cross-link restraints (eq 8 of the main text) for different restraint types (indicated in the respective panels) and the best models obtained from simulations. LR(σ,A) denotes Lorentz-like potentials, with σ and A being the wall thickness and well depth, respectively (eq 8) of the main text, and MD denotes MD-based potentials (eqs 3 – 6) of the main text.

Table S1: Parameters of the expressions for the cross-link virtual-bond potentials (eq 4 of the main text).

Link type	N_d	a_1 $\left[\frac{\text{kcal}}{\text{mol}}\right]$	k_1 $\left[\frac{\text{kcal}}{\text{mol}\times\text{Å}^2}\right]$	d_1° [Å]	a_2 $\left[\frac{\text{kcal}}{\text{mol}}\right]$	k_2 $\left[\frac{\text{kcal}}{\text{mol}\times\text{Å}^2}\right]$	d_2° [Å]	a_3 $\left[\frac{\text{kcal}}{\text{mol}}\right]$	k_3 $\left[\frac{\text{kcal}}{\text{mol}\times\text{Å}^2}\right]$	d_3° [Å]	a_4 $\left[\frac{\text{kcal}}{\text{mol}}\right]$	k_4 $\left[\frac{\text{kcal}}{\text{mol}\times\text{Å}^2}\right]$	d_4° [Å]	a_5 $\left[\frac{\text{kcal}}{\text{mol}}\right]$	k_5 $\left[\frac{\text{kcal}}{\text{mol}\times\text{Å}^2}\right]$	d_5° [Å]
$C^\alpha \cdots X$ and $Y \cdots C^\alpha$ virtual bonds.[a]																
K-DSA-K	3	4.878	23.951	3.421	0.118	3.677	5.386	0.026	248.893	6.342						
C-SDA-K	1	-1.062	119.221	2.778												
	4	0.528	4.170	3.378	0.362	7.505	4.827	0.130	8.000	6.295	-0.008	18.528	5.501			
E-SDA-K	4	0.087	0.549	3.301	-0.637	88.906	4.403	1.424	0.954	11.229	-0.308	95.773	4.910			
	4	1.416	4.462	3.160	1.955	13.188	4.988	0.450	328.066	6.365	0.076	0.947	5.695			
S-SDA-K	1	-1.120	149.222	2.397												
	4	1.537	5.488	3.242	3.728	131.108	4.717	0.885	190.212	6.359	0.009	0.846	5.519			
T-SDA-K	1	-0.994	125.743	2.372												
	5	0.047	0.338	3.100	0.300	1.067	5.385	0.584	10.368	5.499	1.462	109.254	6.366	1.463	109.254	6.366
Y-SDA-K	3	0.063	8.899	3.543	0.677	3.991	5.085	5.352	338.915	6.078						
	5	0.013	0.173	3.288	0.099	1.008	4.960	-0.834	267.420	5.528	0.345	73.545	6.378	3.052	197.621	5.946
$Y \cdots X$ virtual bond																
K-DSA-K	3	4.024	2.819	4.420	0.527	3.578	7.585	1.113	2.486	6.211						
C-SDA-K	3	1.254	8.051	3.808	0.161	5.238	5.551	0.152	133.65	6.453						
E-SDA-K	3	0.348	6.420	3.676	0.426	5.009	5.127	2.406	344.20	6.093						
S-SDA-K	3	0.240	8.604	3.546	0.440	4.373	5.091	2.500	532.23	6.090						
T-SDA-K	3	0.225	9.830	3.583	0.566	5.286	4.810	1.681	21.602	5.702						
Y-SDA-K	3	0.063	8.899	3.543	0.677	3.992	5.085	5.352	338.92	6.078						

[a]Y denotes the lysine-binding site of DSA or SDA and X the photoactive site of SDA. For SDA, the upper rows contain the parameters of the $C^\alpha \cdots Y$ and the lower rows those of the $Y \cdots C^\alpha$ virtual bonds. There is only one row for DSA because this is a symmetric homobifunctional cross-linker.

Table S2: Parameters of the expressions for the cross-link virtual-bond-angle potentials (eq 5 of the main text).[a]

Link type	N_θ	a_1 $\left[\frac{\mathrm{kcal}}{\mathrm{mol}}\right]$	b_1 $\left[\frac{\mathrm{kcal}}{\mathrm{mol}}\right]$	a_2 $\left[\frac{\mathrm{kcal}}{\mathrm{mol}}\right]$	b_2 $\left[\frac{\mathrm{kcal}}{\mathrm{mol}}\right]$	a_3 $\left[\frac{\mathrm{kcal}}{\mathrm{mol}}\right]$	b_3 $\left[\frac{\mathrm{kcal}}{\mathrm{mol}}\right]$	a_4 $\left[\frac{\mathrm{kcal}}{\mathrm{mol}}\right]$	b_4 $\left[\frac{\mathrm{kcal}}{\mathrm{mol}}\right]$	a_5 $\left[\frac{\mathrm{kcal}}{\mathrm{mol}}\right]$	b_5 $\left[\frac{\mathrm{kcal}}{\mathrm{mol}}\right]$	a_6 $\left[\frac{\mathrm{kcal}}{\mathrm{mol}}\right]$	b_6 $\left[\frac{\mathrm{kcal}}{\mathrm{mol}}\right]$
K-DSA-K	6	1.023	-31.686	-218.643	545.730	-3.291	-477.347	71.206	161.151	5.151	-477.221	152.706	279.417
C-SDA-K	6	1.780	102.553	-1.860	-1.366	8.450	-91.394	-3.355	-238.915	214.373	-88.390	302.545	-186.417
	6	0.850	9.608	-2.119	18.194	7.721	-20.336	-8.430	-18.017	14.114	-18.918	90.311	-58.989
E-SDA-K	6	-0.337	-14.236	0.840	25.718	6.814	-2.788	-8.860	32.247	-25.302	3.426	11.383	-13.278
	6	0.830	-42.955	1.451	-12.734	105.396	-86.204	32.928	1.674	15.440	27.162	-67.350	33.616
S-SDA-K	6	3.112	-127.786	-3.590	54.746	6.148	80.283	-13.815	282.097	-227.747	97.139	-307.085	169.366
	6	-0.016	-54.971	1.464	34.646	2.888	26.129	-13.745	136.980	-118.263	34.646	-85.007	45.218
T-SDA-K	6	0.782	-53.511	0.964	36.213	8.990	29.679	-13.050	130.497	-108.275	37.060	-97.629	51.122
	6	0.130	-69.682	1.565	38.874	3.271	37.517	-16.401	175.497	-149.812	47.848	-128.493	71.200
Y-SDA-K	6	2.732	-8.359	-4.157	10.627	-4.036	-6.823	21.681	-7.694	5.119	48.559	-11.901	-36.468
	6	-0.420	-7.854	-19.290	36.958	3.111	-25.041	27.986	9.271	2.615	-14.713	-2.051	1.026

[a]For the Z-SDA-Lys systems, the upper rows are for the $C^\alpha \cdots X \cdots Y$ and the lower rows for the $Y \cdots C^\alpha \cdots X$ virtual-bond angles, where X denotes the amino-acid residue binding to the photoactive site of SDA and Y denotes the lysine-binding site of SDA. For DSA, which is a symmetric homobifunctional cross-linker, both binding sites are equivalent and, consequently, there is only one set of parameters.

Table S3: Parameters of the expressions for the cross-link virtual-bond-dihedral-angle potentials (eq 6 of the main text).

Link type	N_γ	V_1 $\left[\frac{\text{kcal}}{\text{mol}}\right]$	V_2 $\left[\frac{\text{kcal}}{\text{mol}}\right]$	V_3 $\left[\frac{\text{kcal}}{\text{mol}}\right]$
K-DSA-K	2	-0.314	-0.007	
C-SDA-K	2	-0.165	0.013	
E-SDA-K	2	-0.511	-0.069	
S-SDA-K	2	-0.629	-0.059	
T-SDA-K	2	-0.627	-0.051	
Y-SDA-K	3	-0.697	-0.148	-0.071

Table S4: Positions of the synthetic short (SDA- and DSA-type) cross-links of the first benchmark set used in this work to evaluate various cross-linking potentials.

ID	N[a]	N^{b}_{start}	Type	N^{c}_{link}	Cross-link list[d]
1BF0	60	1	β	5 (4)	Y6–K56, C7–K26, K18–S38, K26–C32, E52–K55
1CLB	75	1	α	16(11)	K1–E4, K1–E5, K12–E35, K16–E27, K25–E60, E51–K55, E52–K55, K55–E65, K16–S24, Y13–K16, K1–K71*, K16–K25*, K25–K55*, K29–K41*, K41–K71*, K41–K72*
1CVO	62	1	β	9 (7)	K2–T14, K2–C15, C3–K13, C3–K60, C15–K19, K24–Y53, T26–K37, T26–K52, S48–K52
1E68	70	1	α	6 (4)	K14–K58, T20–K46, E43–K51, K50–K58*, K54–K58*, K55–K58*
1EM7	56	1	$\alpha + \beta$	13(12)	T1–K50, T2–K50, Y3–K50, K4–E15, K4–T17, K4–T49, K4–T51, K10–E56, K13–E56, T17–K50, Y45–K50, K4–K50*, K10–K13*
2FMR	65	1	$\alpha + \beta$	2 (2)	Q7–K59, K31–K59*
1GF4	86	1	$\alpha + \beta$	7 (6)	T13–K22, E25–K28, K42–C48, K42–S49, K42–Y50, C48–K84, S49–K84
1HNS	47	90	$\alpha + \beta$	12(10)	K95–T107, K95–T109, S97–K127, T98–K106, T98–K120, E101–K106, E104–K106, T114–K119, K120–S128, E124–K127, K120–K127*, K127–K135*
1KOY	62	239	α	3 (3)	K248–S254, K248–K281*, K269–K280*
2L09	62	1	α	3 (3)	K12–E46, K10–K25*, K14–K25*
1RXR	84	130	α	6 (3)	C138–K181, K145–Y150, Y147–K201, E153–K156, T162–K165, C190–K194
1TIG	90	83	$\alpha + \beta$	14(13)	E87–K117, S91–K123, T93–K123, K115–E151, K117–T152, K86–K102*, K86–108*, K86–K112*, K86–K115*, K86–K117*, K112–K115*, K112–K168*, K115–K168*, K130–K155*

[a]Number of residues in the PDB structure.

[b]The number of the first residue in the PDB structure.

[c]Number of synthetic cross-links, the long-range cross-links (bridging the residues with indices differing by at least 5) in parentheses.

[d]Residue numbering follows that of the PDB structure. Asterisks mark the DSA-type cross-links, the SDA-type cross-links are unmarked.

Table S5: Positions of the synthetic longer (BS^3) Lys-Lys cross-links for the proteins of the benchmark set of ref 1.

ID	N^a	N^b_{start}	Type	N^c_{link}	Cross-link list^d
1A6S	86	1	α	21 (20)	K6–K13, K6–K18, K13–K18, K13–K23, K13–K24, K13–K67, K13–K72, K18–K23, K18–K24, K18–K82, K23–K24, K23–K35, K23–K67, K23–K72, K24–K35, K24–K67, K24–K72, K35–K67, K35–K72, K67–K72, K72–K82
1BG8	76	10	α	5 (4)	K42–K97, K42–K79, K75–K79, K77–K84, K79–K84
1K40	126	921	α	9 (9)	K933–K941, K933–K955, K933–K1032, K941–K955, K941–K1000, K941–K1017, K955–K1002, K1000–K1017, K1000–K1018
1HRE	67	175	β	6 (4)	K181–K185, K181–K187, K181–K200, K181–K211, K185–K187, K228–K234
1IYU	78	1	β	6 (5)	K21–K53, K21–K58, K39–K62, K48–K53, K58–K62, K58–K64
1UBQ	76	1	$\alpha + \beta$	3 (2)	K6–K11, K11–K33, K29–K33
1VIG	71	6	$\alpha + \beta$	4 (3)	K19–K27, K19–K51, K36–K40, K36–K68

^{a}Number of residues in the PDB structure.

^{b}The number of the first residue in the PDB structure.

^{c}Number of synthetic cross-links between lysine residues, the long-range cross-links (bridging the residues with indices differing by at least 5) in parentheses.

^{d}Residue numbering follows that of the PDB structure.

Table S6: Positions of the experimental cross-links for the four selected repeats of human albumin (1AO6-1, 1AO6-2, 1AO6-3, and 1AO6-6; data from ref 2) and horse myoglobin (2V1H; data from ref 3).

ID	N[a]	N^b_{start}	Type	N^c_{link}	Disulfide bridges (marked SS) and cross-link (marked with the appropriate cross-linking-agent abbreviation) list[d].
1AO6-1	101	5	α	3<br>5 (5)	C53–C62 (SS), C75–C91 (SS), C90–C101 (SS)<br>K12–E57 (DSA), E45–K73 (DSA), E48–K73 (DSA), K93–E100 (DSA), K93–C101 (DSA)
1AO6-2	101	106	α	3<br>5 (5)	C124–C169 (SS), C168–C177 (SS)<br>K137–A161 (DSA), Y138–K162 (DSA), Y148–K199 (DSA), Y150–K199 (DSA), E167–K181 (DSA)
1AO6-3	92	208	α	3<br>3 (3)	C245–C253 (SS), C265–C279 (SS), C278–C289 (SS)<br>K233–Y263 (DSA), E252–K262 (DSA), K281–K292 (DSA)
1AO6-6	84	499	α	2<br>3 (1)	C514–C559 (SS), C558–C567 (SS)<br>K536–T540 (DSA), K538–E542 (DSA), K557–E571 (DSA)
2V1H	153	1	α	20 (20)	G1–K133 (ABAS), K56–K118 (ABAS), G1–G80 (TATA), G1–A130 (TATA), S3–A130 (TATA), T39–I99 (TATA), K45–K98 (TATA), A90–K145 (TATA), G1–K145 (DSA), K87–K147 (DSA), K87–K145 (DSA), K98–K147 (DSA), K42–K98 (DSG), G1–H81 (SDA), V17–K77 (SDA), E18–K77 (SDA), H24–K56 (SDA), Q26–K56 (SDA), E27–K56 (SDA), L29–K42 (SDA)

[a]Number of residues in the PDB structure.

[b]The number of the first residue in the PDB structure.

[c]Number of cross-links, the long-range cross-links (bridging the residues with indices differing by at least 5) in parentheses.

[d]The cross-links corresponding to pairs of residues with the C^α-distances greater by more than 10 Å than the maximum cross-link span are in red font and those with C^α-distances greater by 5–10 Å than the maximum cross-link span are in orange font. All other cross-links are in regular font.

Table S7: C^{α}-RMSD, TM-score, and GDT_TS values of the first and best models of the 12 short-link benchmark proteins obtained in the free and restrained simulations.[a]

ID	Model 1			Best model		
	C^{α}-RMSD	TM-score	GDT_TS	C^{α}-RMSD	TM-score	GDT_TS
Free simulations						
1BF0	10.38	0.2540	29.17	9.73	0.2540	29.17
1CLB	11.09	0.2702	33.00	9.16	0.2875	34.00
1CVO	11.78	0.1765	21.37	9.31	0.1847	25.00
1E68	9.49	0.3510	39.29	5.41	0.3698	46.07
1EM7	5.25	0.3745	54.02	4.80	0.4420	58.04
2FMR	11.01	0.2182	27.69	8.36	0.2795	33.46
1G4F	12.07	0.2195	22.67	11.86	0.2809	28.49
1HNS	10.57	0.2492	37.23	8.28	0.2696	41.49
1KOY	6.55	0.3008	40.73	6.73	0.3438	41.94
2L09	12.57	0.2350	28.23	9.51	0.2556	31.05
1RXR	11.95	0.2828	28.92	11.95	0.2828	28.92
1TIG	11.67	0.2572	26.70	12.58	0.3264	34.38
MD-based restraint potentials						
1BF0	12.16	0.2365	30.00	8.42	0.2748	34.58
1CLB	9.78	0.2334	27.00	8.68	0.2728	29.00
1CVO	8.25	0.2578	34.68	8.25	0.2578	34.68
1E68	8.01	0.3206	36.79	5.22	0.3206	43.57
1EM7	8.44	0.3410	41.52	7.21	0.3821	46.88
2FMR	10.67	0.2548	31.54	9.43	0.2548	31.54
1G4F	11.26	0.2244	24.13	10.46	0.3147	31.40
1HNS	7.03	0.2390	38.83	6.35	0.2485	40.43
1KOY	8.64	0.2782	37.90	6.80	0.3412	39.11

2L09	6.64	0.3638	45.16	6.64	0.3910	45.97
1RXR	11.38	0.2209	24.10	11.29	0.2780	28.31
1TIG	10.01	0.2632	25.57	9.93	0.2728	29.26
Lorentz-like potential, $\sigma = 5$ Å, $A = 8$ kcal/mol						
1BF0	8.85	0.2559	30.42	8.85	0.3062	36.25
1CLB	7.14	0.2726	34.00	7.14	0.3320	37.00
1CVO	8.27	0.2581	33.06	7.88	0.3192	34.68
1E68	4.10	0.4360	50.36	4.10	0.4360	50.36
1EM7	3.15	0.5503	68.75	3.15	0.5503	68.75
2FMR	11.29	0.2367	27.69	8.29	0.2731	34.23
1G4F	11.92	0.2673	28.78	10.75	0.3461	35.47
1HNS	8.54	0.2535	40.43	6.94	0.2725	40.43
1KOY	8.96	0.2798	36.69	8.49	0.3176	36.69
2L09	8.25	0.2943	37.50	6.06	0.4249	47.98
1RXR	12.15	0.2983	31.63	12.15	0.2983	31.63
1TIG	8.19	0.4297	40.06	6.68	0.4297	44.03
Lorentz-like potential, $\sigma = 15$ Å, $A = 8$ kcal/mol						
1BF0	9.17	0.2563	32.92	9.17	0.2563	32.92
1CLB	5.94	0.3554	44.33	5.94	0.3908	44.33
1CVO	10.40	0.1921	25.81	8.45	0.3107	35.48
1E68	6.07	0.2903	38.21	5.97	0.4063	49.29
1EM7	3.27	0.5016	62.50	3.25	0.5409	67.41
2FMR	11.70	0.2091	26.92	9.38	0.2642	33.46
1G4F	11.26	0.3000	29.65	10.74	0.3016	31.10
1HNS	6.60	0.3294	48.94	5.82	0.3681	52.13
1KOY	10.82	0.2533	30.65	6.38	0.3510	43.15
2L09	8.72	0.4126	47.58	8.09	0.3809	47.58

1RXR	10.41	0.2282	24.70	10.41	0.2366	24.70
1TIG	8.86	0.4646	46.31	3.28	0.6217	60.51
Lorentz-like potential, $\sigma = 5$ Å, $A = 20$ kcal/mol						
1BF0	8.78	0.2615	34.17	8.19	0.2870	35.42
1CLB	7.79	0.3306	38.67	7.79	0.3306	38.67
1CVO	8.69	0.2834	35.08	8.03	0.2834	35.08
1E68	5.09	0.3346	43.57	4.76	0.4068	48.93
1EM7	3.88	0.4339	58.93	3.02	0.5556	68.30
2FMR	10.19	0.2137	26.54	7.68	0.2377	30.00
1G4F	9.61	0.3650	36.05	9.61	0.3952	37.21
1HNS	7.77	0.2748	40.43	7.77	0.2748	40.43
1KOY	10.78	0.2154	27.82	6.98	0.3029	38.31
2L09	6.30	0.3899	46.77	5.67	0.3899	48.79
1RXR	8.77	0.2575	27.11	8.77	0.2802	28.61
1TIG	7.08	0.3621	37.50	6.86	0.4015	40.62
Lorentz-like potential, $\sigma = 15$ Å, $A = 20$ kcal/mol						
1BF0	7.99	0.2657	32.92	7.99	0.2657	32.92
1CLB	4.66	0.4402	47.67	4.65	0.4402	47.67
1CVO	8.89	0.2388	29.03	7.58	0.2532	30.65
1E68	4.30	0.4489	52.50	4.08	0.4517	53.57
1EM7	3.10	0.5422	67.41	3.10	0.5410	67.41
2FMR	10.28	0.2005	24.62	7.81	0.2834	33.85
1G4F	13.44	0.2344	25.58	8.93	0.3712	36.05
1HNS	7.99	0.2474	36.70	7.41	0.3342	43.62
1KOY	10.25	0.2524	33.87	6.84	0.3194	40.73
2L09	8.02	0.3188	39.52	6.68	0.3824	43.95
1RXR	11.96	0.1931	19.58	11.96	0.2227	23.49

1TIG	6.94	0.5446	52.27	5.10	0.5446	52.27

[a]The number of significant digits of GDT_TS and C^{α}-RMSD values follows the convention of reporting these values in the CASP experiments (https://www.predictioncenter.org). Following this convention, TM-score values, which range from 0 to 1, are reported with 4 digits after the decimal separator.

Table S8: C^{α}-RMSD, TM-score, and GDT_TS values of the first and best models of the 7 benchmark proteins from ref 1 obtained in free and cross-link-restrained of simulations.[a]

ID	Model 1			Best model		
	C^{α}-RMSD	TM-score	GDT_TS	C^{α}-RMSD	TM-score	GDT_TS
Free simulations[b]						
1A6S	10.94	0.1810	20.64	10.57	0.2332	25.00
1BG8	12.56	0.2390	25.99	10.51	0.2462	25.99
1K40	15.00	0.2260	19.25	13.63	0.2663	22.42
1HRE	14.31	0.1937	21.64	14.31	0.1937	23.88
1IYU	10.18	0.2301	25.96	10.18	0.2301	25.96
1UBQ	11.41	0.2252	26.32	9.20	0.3101	34.87
1VIG	10.40	0.2556	29.58	9.44	0.2556	29.58
Knowledge-based C^{α}-restraint potentials[b]						
1A6S	10.31	0.2356	26.74	9.77	0.2356	26.74
1BG8	11.61	0.2106	25.00	9.36	0.2932	32.24
1K40	11.17	0.2917	25.79	7.03	0.3840	36.31
1HRE	13.49	0.1927	23.13	13.15	0.2215	27.24
1IYU	11.71	0.2055	24.68	10.23	0.2451	25.32
1UBQ	13.14	0.1970	24.01	10.18	0.2724	30.59
1VIG	9.62	0.2171	26.76	7.17	0.3085	39.08
MD-based restraint potentials[b]						
1A6S	8.77	0.2778	29.02	8.77	0.2874	29.02
1BG8	11.24	0.2035	23.03	9.03	0.2279	26.64
1K40	8.99	0.3491	29.56	8.99	0.3491	29.56
1HRE	13.71	0.1769	20.90	11.59	0.2062	24.63
1IYU	12.05	0.1926	23.40	10.77	0.2538	25.96
1UBQ	9.56	0.2723	31.25	9.54	0.2909	33.88
1VIG	10.11	0.2869	34.86	8.06	0.2869	35.92
Lorentz-like potential, $d_u = 12$ Å, $\sigma = 5$ Å, $A = 8$ kcal/mol						
1A6S	9.68	0.3176	31.61	7.40	0.3176	33.05
1BG8	10.84	0.2160	25.00	10.18	0.2681	30.26
1K40	12.34	0.2502	21.43	11.74	0.3751	31.94
1HRE	12.34	0.2027	23.13	13.09	0.2167	28.73
1IYU	12.88	0.2065	23.42	11.96	0.2108	23.42
1UBQ	10.67	0.2938	34.87	6.81	0.4171	44.74
1VIG	11.00	0.2309	27.11	9.02	0.2434	30.63
Lorentz-like potential, $d_u = 12$ Å, $\sigma = 15$ Å, $A = 8$ kcal/mol						
1A6S	10.14	0.2709	30.17	8.66	0.3331	35.63

1BG8	12.14	0.2171	24.01	10.31	0.2646	31.58
1K40	11.42	0.3088	26.98	10.96	0.3819	32.34
1HRE	13.72	0.1964	23.88	13.21	0.2017	23.88
1IYU	10.33	0.2423	26.27	10.33	0.2791	29.11
1UBQ	11.41	0.2528	30.26	10.66	0.2936	32.24
1VIG	9.49	0.3004	36.62	9.41	0.3004	36.62
Lorentz-like potential, $d_u = 12$ Å, $\sigma = 5$ Å, $A = 20$ kcal/mol						
1A6S	9.20	0.3024	32.18	9.20	0.3024	32.18
1BG8	11.43	0.2432	28.62	10.61	0.2510	29.61
1K40	10.67	0.3456	29.17	10.67	0.3456	29.17
1HRE	16.18	0.1754	21.27	11.92	0.2097	24.63
1IYU	11.47	0.2496	28.80	8.97	0.2591	29.43
1UBQ	11.11	0.3115	33.88	10.27	0.3265	35.20
1VIG	9.92	0.2421	26.06	9.74	0.2411	28.17
Lorentz-like potential, $d_u = 12$ Å, $\sigma = 15$ Å, $A = 20$ kcal/mol						
1A6S	10.20	0.2673	28.45	9.38	0.269	28.45
1BG8	9.37	0.3012	32.57	9.37	0.3012	32.57
1K40	3.92	0.6234	52.98	3.92	0.6234	52.98
1HRE	13.92	0.2050	22.39	12.58	0.2714	30.60
1IYU	10.78	0.2638	26.27	10.78	0.2638	28.80
1UBQ	8.92	0.3685	39.80	8.92	0.3685	39.80
1VIG	9.04	0.2578	28.52	8.80	0.2578	30.63

[a]The number of significant digits of GDT_TS and C^{α}-RMSD values follows the convention of reporting these values in the CASP experiments (https://www.predictioncenter.org). Following this convention, TM-score values, which range from 0 to 1, are reported with 4 digits after the decimal separator.

[b]Data from ref 1.

Table S9: C^{α}-RMSD, TM-score, and GDT_TS values of the first and best models of the first (1AO6-1), second (1AO6-2), third (1AO6-3), and sixth (1AO6-6) repeat of human serum albumin and myoglobin (2V1H) benchmark systems, for which experimental cross-link data were used.[a]

ID	Model 1			Best model		
	C^{α}-RMSD	TM-score	GDT_TS	C^{α}-RMSD	TM-score	GDT_TS
Dihedral-angle and disulfide-bridge restraints (for 1AO6-1 – 1AO6-6 only)[b]						
1AO6-1	10.57	0.4420	41.83	7.47	0.4492	42.33
	6.75	0.4830	45.54	5.65	0.5324	50.50
1AO6-2	9.90	0.4555	42.16	7.91	0.4555	42.16
	10.62	0.4949	47.79	5.65	0.5219	50.74
1AO6-3	8.98	0.5030	48.61	8.10	0.5266	51.94
	5.00	0.4850	48.89	5.00	0.5169	50.83
1AO6-6	16.27	0.3307	36.01	12.57	0.3670	39.29
	12.25	0.3525	38.99	10.62	0.3720	42.26
2V1H	9.23	0.3878	30.39	9.23	0.3878	30.39
Lorentz-like restraints, $\sigma = 5$ Å, $A = 8$ kcal/mol						
1AO6-1	6.27	0.4212	43.56	5.12	0.4897	48.27
1AO6-2	6.72	0.6041	55.64	5.99	0.6041	55.64
1AO6-3	4.76	0.4643	48.33	4.76	0.5338	52.22
1AO6-6	12.84	0.4247	46.13	10.05	0.4247	46.13
2V1H	4.29	0.6130	46.57	4.29	0.6130	46.57
Lorentz-like restraints, $\sigma = 5$ Å, $A = 20$ kcal/mol						
1AO6-1	4.70	0.5574	51.49	4.70	0.5574	51.49
1AO6-2	7.22	0.5636	54.90	6.10	0.5636	54.90
1AO6-3	5.58	0.4282	43.33	5.17	0.4988	50.28
1AO6-6	12.26	0.4907	50.00	12.12	0.4907	50.00
2V1H	7.50	0.4317	32.68	6.94	0.5065	37.91
Lorentz-like restraints, $\sigma = 15$ Å, $A = 8$ kcal/mol						
1AO6-1	6.88	0.3991	40.35	5.36	0.4979	47.77
1AO6-2	6.90	0.5835	54.90	6.20	0.6457	58.58
1AO6-3	8.63	0.4479	46.39	5.37	0.5372	53.61
1AO6-6	12.23	0.3881	43.45	7.53	0.3986	43.45
2V1H	3.90	0.6601	55.07	3.89	0.6601	55.07
Lorentz-like restraints, $\sigma = 15$ Å, $A = 20$ kcal/mol						
1AO6-1	5.69	0.4104	43.07	5.69	0.4781	45.30
1AO6-2	6.59	0.4993	47.06	5.96	0.6032	54.90
1AO6-3	7.50	0.3845	38.89	4.55	0.5730	56.11

1AO6-6	12.34	0.4146	44.05	10.53	0.4146	44.05
2V1H	3.92	0.6502	53.27	3.92	0.6502	53.27
MD-derived potentials[c]						
1AO6-1	5.55	0.4618	46.78	5.55	0.4898	50.99
1AO6-2	10.95	0.4603	43.38	6.15	0.5116	48.53
1AO6-3	5.82	0.4418	44.17	5.25	0.4481	46.11
1AO6-6	12.35	0.3470	40.18	8.29	0.4284	44.05

[a]The number of significant digits of GDT_TS and C^{α}-RMSD values follows the convention of reporting these values in the CASP experiments (https://www.predictioncenter.org). Following this convention, TM-score values, which range from 0 to 1, are reported with 4 digits after the decimal separator.

[b]Upper line for 11AO6-1, 1AO6-2, 1AO6-3, and 1AO6-6: dihedral-angle restraints only; lower lines: dihedral-angle and disulfide-bridge restraints.

[c]MD-derived potentials are not available for most of the cross-links reported for myoglobin (2V1H); therefore the respective calculations were not performed.

References

(1) Kogut, M.; Gong, Z.; Tang, C.; Liwo, A., Pseudopotentials for Coarse-Grained Cross-Link-Assisted Modeling of Protein Structures. *J. Comput. Chem.* **2021**, *42*, 2054–2067.

(2) Belsom, A.; Schneider, M.; Fischer, L.; Brock, O.; Rappsilber, J., Serum Albumin Domain Structures in Human Blood Serum by Mass Spectrometry and Computational Biology. *Mol. Cell. Proteomics* **2016**, *15*, 1105–1116.

(3) Brodie, N. I.; Popov, K. I.; Petrotchenko, E. V.; Dokholyan, N. V.; Borchers, C. H., Solving Protein Structures Using Short-Distance Cross-Linking Constraints as a Guide for Discrete Molecular Dynamics Simulations. *Sci. Adv.* **2017**, *3*, e1700479.
